# Untargeted metabolomics suggests a trade-off between phenolamides and flavonoids in the pollen of *Petunia hybrida* with contrasting flower colours

**DOI:** 10.1186/s12870-026-08968-y

**Published:** 2026-06-11

**Authors:** Matheus Fernandes Alves, Abigail Moreno-Pedraza, Paula Carolina Pires Bueno, Khabat Vahabi, Nicole M. van Dam

**Affiliations:** 1 Institute of Biodiversity, Ecology, and Evolution (IBEE), Friedrich Schiller University Jena, Dornburgerstraße 159, Jena, 07743 Germany; 2 Leibniz Institute of Vegetable and Ornamental Crops (IGZ), Theodor-Echtermeyer-Weg 1, Großbeeren, 14979 Germany; 3 Earth and Biological Sciences Directorate, Pacific Northwest National Laboratory, Richland, Washington 99354 USA

**Keywords:** *Petunia hybrida*, Pollen metabolomics, Pollen analysis, Metabolic profiling, Liquid chromatography - mass spectrometry, Specialised metabolites, Phenylpropanoids

## Abstract

**Background:**

Pollen is vital for reproduction of flowering plants. Several nutritional and biological benefits for humans and pollinators are described and related to its richness and diversity of metabolites. However, the chemical composition of pollen from several horticulturally significant plant species, such as those in the genus *Petunia*, has not been thoroughly characterised. Here, we present the first comprehensive description of the chemical profile of two distinct *P. hybrida* lines: V26 (violet flowers) and W115 (white flowers) using untargeted metabolomics. Our workflow started from pollen sampling and isolation, followed by a 3-in-1 liquid phase extraction for wide-range metabolite recovery, and data acquisition by ultra-high-performance liquid chromatography coupled to high-resolution tandem mass spectrometry (UHPLC-HRMS/MS) using three chromatographic columns (C8, C18, and HILIC) with positive and negative ionisation. For data analysis, we implemented a user-friendly and reproducible data processing pipeline based on open-source computational tools.

**Results:**

The *P. hybrida* pollen is rich in glycosylated flavonoids, phenolamides, and lipids, which were detected mostly in non-polar phase extracts analysed by reversed-phase chromatography. Simple phenylpropanoids, fatty acids, amino acids, and terpenoids were annotated to a lesser extent. Statistical analyses integrated with molecular networking demonstrated a distinct metabolic dichotomy: phenolamide derivatives are predominantly present in the pollen of the V26 line, while flavonoids are accumulated in the pollen of W115. This finding suggests different regulation of the phenylpropanoid metabolism in pollen of *P. hybrida* lines with differing flower colours and sheds light on hypotheses of ecological roles of pollen secondary metabolites, for example, in plant-pollinator interactions.

**Conclusions:**

Our findings suggest a metabolic trade-off in the phenylpropanoid pathway in the two studied *P. hybrida* lines. These cultivar-specific chemical signatures may have significant implications for pollen viability and interactions with pollinators. Furthermore, our analytical and computational workflow serves as a robust template for the deep metabolic profiling of other under-characterised and complex natural matrices.

**Supplementary Information:**

The online version contains supplementary material available at 10.1186/s12870-026-08968-y.

## Background

Pollen is a cornerstone element of terrestrial life, playing an essential role in the reproduction of flowering plants, whose success is intrinsically linked to the biological viability of pollen grains. In addition, pollen serves as essential nutrition for a wide array of pollinators and insects, thereby underpinning an important resource for terrestrial ecosystems [1, 2]. Both pollen viability and nutritional value to pollinators are linked to their molecular and chemical properties [3]. Previous studies have shown that pollen grains possess a wide set of secondary metabolites, such as flavonoids, phenolamides (or hydroxycinnamic acid amides, HCAA), lipids, terpenoids, and alkaloids [4, 5]. These compounds, also called specialised metabolites, are structural or physiological components important in abiotic defence and can contribute to biotic interactions with pollinators, pollen feeders, and microbes [6, 7]. Certain plant species produce pollen containing compounds that can be harmful to generalist insect pollinators and other pollen feeders lacking specific detoxification mechanisms [8]. On the other hand, compounds in pollen can also serve as floral rewards for insects; high levels of the amino acid proline in pollen enhance the reproductive success of queen honeybees [9], and its presence in nectar is regarded as a metabolic reward for flying pollinators [10]. Proline, and gamma-amino butyric acid (GABA), are also important in pollen viability and germination, supporting pollen tube growth, pollen development, and pollination success [11]. Consequently, understanding the chemical composition of pollen and its metabolic diversity is critical for determining how pollen vitality fluctuates and its role in shaping ecological interactions.

Over the last decades, ultra-high-performance liquid chromatography coupled to high-resolution tandem mass spectrometry (UHPLC-HRMS/MS)-based metabolomics has become the gold standard analytical technique for exploring the chemical diversity of complex biological systems [12]. Metabolomics has played an important role in plant biology, supplementing plant functional genomics and opening new perspectives on plant biochemistry [13]. Several studies have successfully employed this approach to investigate different aspects of pollen, such as fermentation effects [14], metabolic profile [15], bioactive compounds [16], and heat stress responses [17, 18]. However, the chemical profile of pollen from several horticulturally significant plant species remains unexplored. This applies to species within the genus *Petunia* (Solanaceae), which is a model organism in plant genetics and metabolic pathway studies [19–21]. In addition, *Petunia* species are among the most famous and sold ornamental plants, with a total sales volume of more than $ 230 million in the USA in 2019 [22].

Modern *P. hybrida* cultivars are derived from early 19th-century crosses between *P. axillaris* (white flower, hawkmoth-pollinated) and *P. integrifolia* species (violet flower, bee-pollinated), resulting in hybrids with normal diploid offspring. *Petunia* laboratory lines such as V26, characterised by its violet flowers, and W115 (also called Mitchell), with white flowers, are easy to transform and yield many plants per experiment [23, 24]. Hybrid lines have been used for over two decades to study the genetics and biosynthetic pathways underlying flower development and coloration [25–27], and are suitable models to study differences in pollen chemistry between flower morphs. Previous studies have described the volatile composition of W115 and its role in pollinator attraction [28, 29], as well as the regulation of flavonoid biosynthesis in V26 pollen [30]. However, the non-volatile metabolome of *Petunia* pollen has not been fully established to date.

**Fig. 1:**
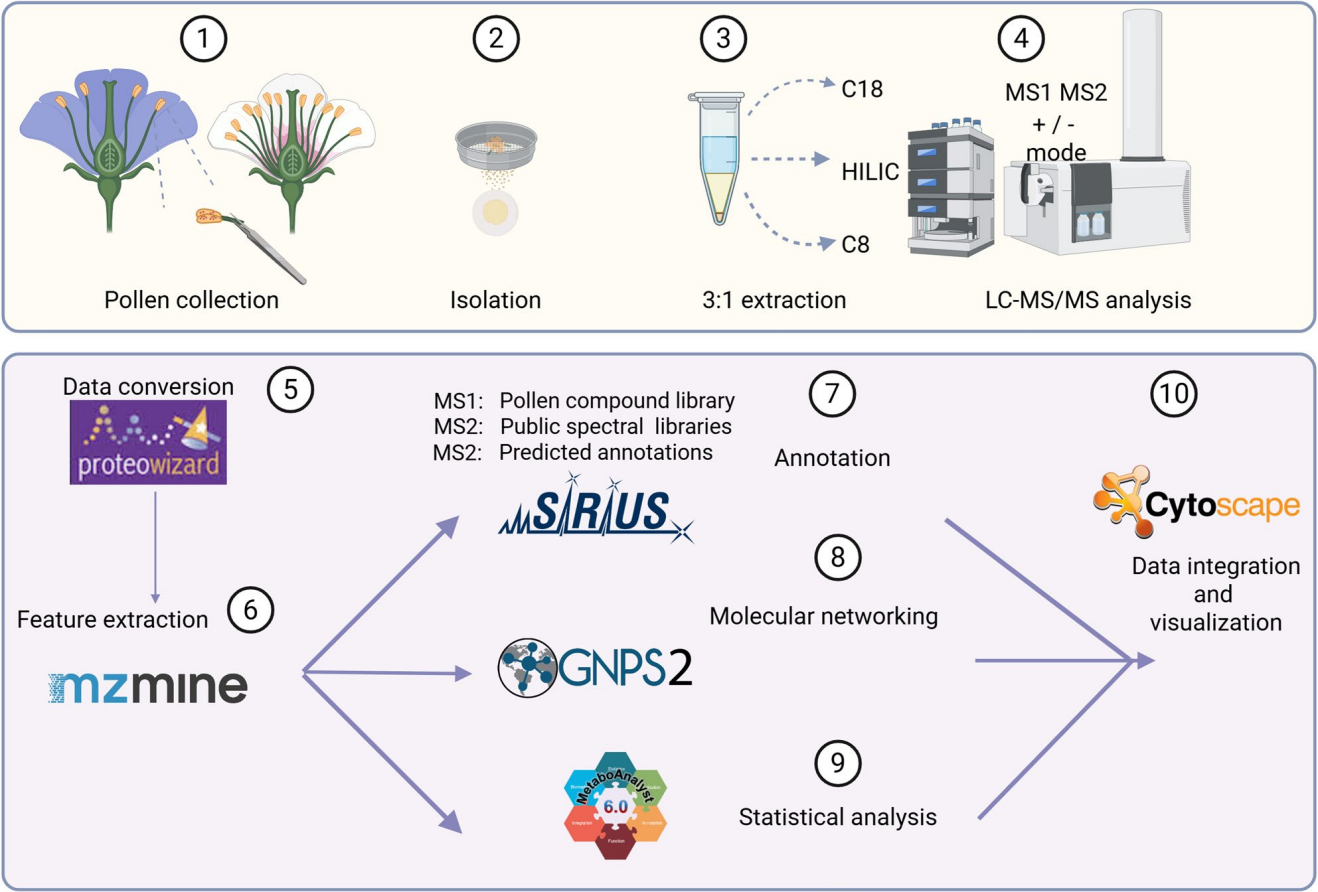
Schematic representation of the untargeted metabolomics workflow for pollen analysis. Steps include (1) sample collection, (2) pollen isolation, (3) metabolite extraction, (4) data acquisition, (5) file conversion, (6) feature extraction, (7) annotation, (8) molecular networking, (9) statistical analysis, and (10) data integration. Created in BioRender. Moreno-Pedraza, A. (https://BioRender.com/i9h13wg)

In this study, we chemically characterised the mature pollen from *Petunia x hybrida* hort. ex E. Vilm (NCBI: txid4102) lines V26 and W115 using UHPLC-HRMS/MS-based untargeted metabolomics. Herein, we established a methodological workflow for pollen collection and isolation, metabolite extraction, application of different chromatographic columns, and data processing, including annotation strategies and statistical analysis (Fig. 1). We used a wet pollen isolation method adapted to the morphology of *Petunia* anthers, followed by a triphasic solvent extraction for recovery of metabolites with different polarities. For chemical analysis, we employed three different chromatographic columns for the separation of non-polar, semi-polar and polar metabolites, electrospray ionisation (ESI) source operating in positive and negative modes, and a quadrupole time-of-flight (QTOF) mass analyser. Data processing and analyses were carried out using open-source software and platforms: MSConvert for data conversion and MZmine for feature extraction (*m*/*z*, retention time - RT, peak area, and fragmentation data - MS2). Putative metabolite annotation was achieved using an *in-house* pollen compound library, SIRIUS for in-silico annotation, and the Global Natural Products Social Molecular Networking (GNPS2) for data organisation and spectral annotation. MetaboAnalyst was used for statistics and Cytoscape for data integration (Fig. 1).

## Methods

### Chemicals

LC-MS grade acetonitrile, methanol, chloroform, and water were purchased from Merck KGaA (Darmstadt, DE), and LC-MS grade formic acid was purchased from Tokyo Chemical Industry Co. (Tokyo, JP). The chemical standards ampicillin (C_16_H_19_N_3_O_4_S, MW 349.40 Da, ≥ 95.0%), corticosterone (C_21_H_30_O_4_, MW 346.46 Da, ≥ 98%), and phosphatidylcholine (PC) 17:0/17:0 (C_42_H_84_NO_8_P, MW 762.09 Da, ≥ 99%) were purchased from Sigma-Aldrich, Co. (St. Louis, MO, USA).

### Plant material and growth conditions

Greenhouse experiments were conducted at the Leibniz Institute of Vegetable and Ornamental Crops (IGZ) located in Großbeeren, Brandenburg, Germany (52°22′N, 13°18′E, alt. 40 m) (Figure S1). *P. hybrida* W115 and V26 seeds were generously provided by Dr. Janny L. Peters of Radboud University Netherlands. Seeds were germinated in designed germination beds. After 10 days, the seedlings were transplanted into individual 2 L pots using a standard soil mix ED73 (Einheitserde Werkverband e.V., DE). Plants were fertilised once a week using a nutrient solution according to De Kreij et al. [31]. Plants were cultivated in glasshouses under natural daylight conditions at 22/18°C day/night and 60% relative humidity; supplemental lighting was provided by Philips IP65 lamps equipped with Master Agro 400 W (Koninklijke Philips N.V., Amsterdam, NL) between 6 a.m. and 6 p.m.

### Anther collection and pollen isolation

Flowers were harvested from July to September 2023 between 9 and 10 a.m. (Figure S1). Fully open flowers were selected, detached, and collected in a clean plastic tray. The flowers were transferred to the laboratory and then carefully dissected using precision stainless tweezers. Anthers from several flowers were collected into 50-mL Falcon tubes, where each biological replicate (1 sample) consisted of anthers collected from approximately 100 flowers. In total, 15 pooled biological replicates, consisting of about 100 flowers each, were prepared per group (V26 and W115). The Falcon tubes containing the anthers were kept at -4 °C for a maximum of 4 days before the pollen isolation.

Pollen isolation requires meticulous physical manipulation and minimised use of solvents. We employed an adapted wet-sampling protocol based on Paupière et al. (2019) [32], which is suitable for *Petunia* flower morphology. Initially, pollen was released from the anthers by vortexing for 1 min with ice-cold distilled water at a 3:1 water: anthers ratio, followed by a two-step filtration process. In the first filtration step, anthers were removed using a 500-µm stainless steel mesh, repeating this step two times. The second filtration step involved vacuum filtration using a 5-µm pore size nylon filter (Merck Millipore, IRL). The resulting filtrate and the pollen in the nylon filter were re-dissolved using 1 mL of ice-cold distilled water, transferred into new 2 mL Eppendorf tubes, and centrifuged at 10,000 rpm (11,292 rcf) for 10 min at 4 °C (5430 R centrifuge, Eppendorf SE, DE). The supernatant was removed and the pellet stored at -70 °C for subsequent lyophilisation.

### Metabolite extraction

We employed a 3-in-1 phase-separation extraction protocol adapted from Wang et al. (2022) [33] to recover compounds of different polarities from a single sample. Briefly, 350 µL of ice-cold methanol: chloroform (70:30, *v*/*v*) spiked with ampicillin, corticosterone, and PC 17:0/17:0 at 25 µg/mL (internal standards) was added to each 2 mL Eppendorf tube containing 5 mg of lyophilised pollen. We used internal standards to evaluate the standard deviation within the samples, RT shift, peak shape deterioration, and mass accuracy error [34]. In addition, two 2-mm stainless steel beads were added to each sample tube and kept cold using ice bath. Cell lysis was conducted using a mixer mill MM 400 (Retsch GmbH, DE) for 2 min at 30 Hz, followed by 10 min of sonication at 35 kHz (SONOREX SUPER RK Bandelin electronic GmbH & Co. KG, DE). During sonication, the temperature was maintained between 10 and 15 °C using gel ice packs to minimise metabolic degradation. Subsequently, 150 µL of ultra-pure water was added to each homogenised extract. Samples were then centrifuged at 15,000 rpm (25,407 rcf) for 15 min and 4 °C (5430 R centrifuge, Eppendorf SE, DE). The samples formed a heterogenous mixture consisting of hydromethanolic and organic phases, which were separated and transferred into new tubes; the hydromethanolic phase divided between two tubes, for hydrophilic interaction chromatography (HILIC) and C18 analyses, and the organic phase into one tube for C8 analyses.

Samples were dried using a vacuum concentrator (Savant SpeedVac SPD130DLX, Thermo Fisher Scientific, EUA). The hydromethanolic phase samples were reconstituted using methanol: water in 70:30 (*v*/*v*) and 30:70 (*v*/*v)* ratios for C18 and HILIC analyses, respectively. For the organic phase, samples were reconstituted in a solvent mixture of acetonitrile: methanol in a 50:50 (*v*/*v)* ratio. The samples were centrifuged twice at 15,000 rpm (25,407 x g) for 10 min at 4 °C to avoid the filtration step common before the LC analyses and minimise material loss, critical for low quantities of biological material [35]. Following centrifugation, 150 µL of each resulting supernatant was transferred into new 2 mL glass vials equipped with 250 µL inserts.

### UHPLC-HRMS/MS data acquisition

The extracts were analysed using an Agilent 1290 Infinity UHPLC coupled to an Agilent 6546 mass spectrometer, with Dual AJS ESI source and QTOF mass analyser (Agilent Technologies, CA, USA). Three chromatographic columns were employed: C8 Agilent Eclipse (3 × 100 mm, 1.8 μm), C18 Agilent Poreshell 120 (2.1 × 150 mm, 1.9 μm) and Agilent HILIC-Z (2.1 × 150 mm, 2.7 μm) (Agilent Technologies, CA, USA). For each column, a chromatographic method was developed and optimised as described in Table S1.

For all analyses, HRMS data were acquired in both positive and negative electrospray ionisation (ESI) modes using data-dependent acquisition (DDA). Acquisitions performed with the C8 and C18 columns employed identical instrumental parameters, as described: capillary voltage = ± 4.5 kV, nozzle voltage = 0 V, fragmentor = 120 V, skimmer = 65 V, and the Octopole RFPeak = 750 V; drying gas temperature = 220 °C, drying gas flow = 8 L/min, nebuliser pressure = 35 psi, sheath gas heater = 240 °C, and sheath gas flow = 12 L/min. Full mass scan (MS1 and MS2) acquired from 90 to 1600 *m*/*z*. DDA experiments were set with a maximum of 3 precursor ions for collision-induced dissociation (CID) at energies of 10 and 30 V, at an acquisition rate of 5 spectra/s. For the acquisition using HILIC, the capillary voltage was = ± 3.5 kV, nozzle voltage = 0 V, fragmentor = 100 V, skimmer = 65 V, and the Octopole RFPeak = 750 V; drying gas temperature = 280 °C, drying gas flow = 8 L/min, nebuliser pressure = 35 psi, sheath gas heater = 300 °C, and sheath gas flow = 12 L/min. Full mass scan (MS1 and MS2) from 40 to 1000 *m*/*z*. DDA experiments were set with a maximum of 3 precursor ions for CID at energies of 15 and 25 eV, at an acquisition rate of 3 spec/s. Throughout all acquisitions, the intensity threshold was set at 200, number of precursors equal to 2, active exclusion released after 0.5 min, instrument resolution (FWHM) of 30,000 at *m/z* 118. Accuracy of *m*/*z* values during data acquisition was monitored by continuous recalibration of the mass axis using reference ions: *m*/*z* 121.0509 and *m*/*z* 922.0080 in positive mode, and *m*/*z* 112.9856 and *m*/*z* 966.0007 in negative mode.

In addition to the samples (*n* = 30), 5 pooled quality control (QC) and 5 blanks were injected between the samples. The QC was prepared by combining 2 µL of each pollen extract into a new 2 mL glass vial equipped with a 200 µL insert, resulting in a final volume of 60 µL. Blanks consisted of resuspension solutions without internal standards. In total, 240 chromatographic runs were performed across the three different chromatographic columns and ionisation modes.

### Data processing – File conversion and feature extraction

Data processing was performed using freely available software and platforms (Table S2). The UHPLC-HRMS/MS data was converted to mzML format using MSConvert (v 3.0.24213) from Proteowizard [36]. The parameters set are summarised in Table S3. We advocate the use of the community standard mzML format for data sharing and interoperability with open software [37]. The converted dataset is available at Zenodo. For additional information on data conversion: https://mzmine.g.ithub.io/mzmine_documentation/data_conversion.html and https://proteowizard.sourceforge.io/tools/msconvert.html.

After data conversion, the six datasets were imported into MZmine (v 4.7.29) for feature extraction through a sequential and modular workflow [38]. Details about modules, algorithms and parameters are available at https://mzmine.g.ithub.io/mzmine_documentation/index.html. The processing steps included: mass detection, chromatogram construction, resolving (deconvolution), deisotoping, alignment, gap filling, adduct search, QC-filtering, and blank subtraction. Each dataset was processed independently. After feature extraction and alignment, the results - spectral data in mascot generic format (MGF) and feature lists in comma separate values (CSV) files - were exported for further annotation and data analysis. The processing modules and parameters utilised are summarised in Table S4-S6. Batch files for data processing, metadata, and resulting feature lists are available at Zenodo.

### Statistical analysis

We utilised Metaboanalyst 6.0 (https://www.metaboanalyst.ca/) for metabolomics data analysis [39]. Feature lists from MZmine were imported using the “statistical analysis [one factor]” module. Data underwent additional processing steps: missing value imputation using a left-censored method with a limit of detection (LOD) set to 1/5 of the minimum positive value for each feature; normalisation by the total ion current (normalisation by sum) to correct analytical inter-sample variation; logarithmic transformation (log_10_) to ensure normal distribution; and scaling using Pareto algorithm to prevent unequal influence of high-abundance features without excessively amplifying noise [40].

The processed datasets were first assessed using Principal Component Analysis (PCA) including the QC group, followed by univariate Student’s t-test for two-group (V26 x W115) comparisons using the Benjamin-Hochberg method to control the false discovery ratio (FDR) and avoid type I errors (false positives) [41, 42]. Features were considered significant based on FDR-adjusted p-value threshold of 1 × 10^− 5^ (*p* ≤ 0.00001) and visualised using Volcano plots alongside a fold change (FC) threshold of 2. The resulting files from PCA, t-test, and Volcano plot are available at Zenodo. Finally, Pearson correlation was performed using the most important features as identified in the Volcano plots and visualised as heatmaps to evidence metabolic correlations.

### Annotation

A combined strategy was employed for annotating features: MS2-based annotation using open spectral libraries; in-silico prediction of chemical classes using MS2 data; and monoisotopic mass search using a compound library (MS1).

MS2-based annotation was performed using the spectral search module in MZmine and publicly available spectral libraries from the MassBank of North America (MoNA) [43] and from the MS-DIAL metabolomics MSP Spectral Kit [44] (Text S1). The parameters for the spectral library search are described in Table S5. Lipid annotation was performed using the module included in Mzmine, searching across all available lipid classes using a *m*/*z* tolerance of 0.005 or 5 ppm. MS1-based annotation was accomplished using the local compound database search module in MZmine and a customised library of previously reported metabolites specific to pollen samples (parameters are listed in Table S6). The customised pollen compound library is available at Zenodo. MS2-based annotations were supplemented with the GNPS libraries through a feature-based molecular networking (FBMN) workflow within GNPS2 platform (https://gnps2.org/homepage, release 2025.67.17) for spectral data organisation [45, 46]. The FBMN parameters and task links are described in Tables S7 and S8.

The compound hits obtained from both MS1 and MS2 annotations were combined and submitted to an AI-assisted data curation using the publicly available Gemini Pro model (Google, CA, USA) via Google AI Studio (https://aistudio.google.com/). The model was prompted to perform a sequential, multi-step analysis for each unique annotation: data cleaning, which involved parsing the raw list and consolidating unique compound names; chemical or biosynthetic classification; biological plausibility assessment, considering the context of the annotation within *P. hybrida* pollen extracts; and classification into categories such as “highly plausible”, “possible but requires scrutiny”, and “nonsensical/false positive”. After filtering the nonsensical annotations (synthetic compounds, artefacts, and non-related natural products), the AI-curated annotation list was manually inspected. The prompt used for AI-assisted review is described in Text S2.

Significant features without library hits were manually annotated using chemical expertise based mainly on MS/MS fragmentation patterns. Flavonoid annotations were guided by aglycone diagnostic ions, whereas phenolamides were assessed via characteristic neutral losses (-cinnamoyl, -coumaroyl, -caffeoyl). Isomers were discriminated based on the RT and assigned as such. Where possible, structural information (molecular formula, possible substituents, and structural core) was inferred, e.g., the aglycone for flavonoids and the polyamine core for phenolamides. Those annotations were assigned as level 3 confidence according to the Metabolomics Standards Initiative (MSI) classification.

The open software SIRIUS (v 6.3.0) was used for in-silico MS2 class predictions. The metabolite chemical classes were determined using structural prediction algorithms: ZODIAC, CANOPUS, CSI: FingerID [47–49]. Specifically, CANOPUS (class assignment and ontology prediction using mass spectrometry) predicted chemical classes from MS2 independent of spectral database, suitable for unknown compounds [49]. Detailed information about SIRIUS: https://v6.docs.sirius-ms.io/methods-background/. Table S9 summarizes the parameters used. The input MGF files for SIRIUS and results are available at Zenodo.

### Data integration

FBMNs exported from GNPS2 were uploaded into the software Cytoscape (v 3.10.3) for data organisation and network visualisation [50]. Results from feature extraction, annotation, and statistical analyses were formatted as CSV files and integrated into Cytoscape alongside the FBMNs, ensuring the feature identification number (ID) from MZmine assigned as the ‘key column’ for integration of each result file. Files for data integration, customised networks, and the network style used are available at Zenodo.

## Results

### Data processing and annotation

**Fig. 2:**
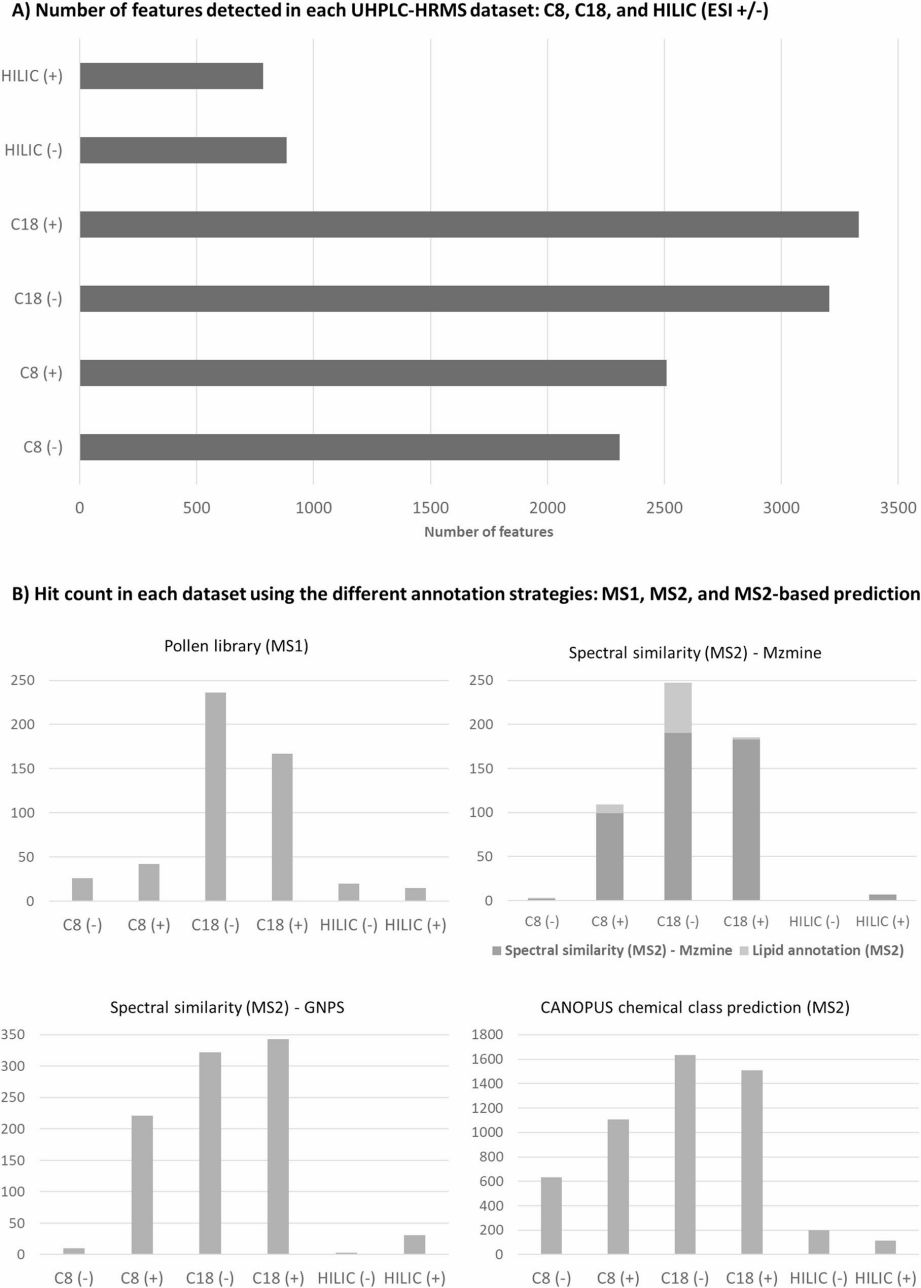
UHPLC-HRMS/MS data processing result summary. **A** Number of the features detected in *Petunia hybrida* pollen extracts analysed using three different chromatographic columns (C8, C18, and HILIC) and two ionisation modes: (+) positive; (-) negative. **B** Total number of annotated compounds: MS1-based annotation using theoretical pollen database; MS2-based spectral similarity search performed at MZMine using public libraries; MS2-based spectral similarity search performed at GNPS; and MS2-based CANOPUS class predictions

The number of chemical features obtained from data processing in MZmine varied depending on the extraction, column, and ionisation mode (Fig. 2A). Non-polar and semi-polar phase extracts analysed using reversed phase chromatography presented complex chromatograms (Figure S2 and S3) and yielded the highest number of features, with more than 3000 using the C18 column, and over 2000 using the C8 column. In contrast, the polar phase extracts analysed using the HILIC column yielded relatively simpler chromatograms (Figure S4) and lower feature count (< 1000).

To provide a chemical profile of each extract, we applied a mixed annotation strategy to generate a broad set of candidate compounds (Fig. 2B) and foundation for biological interpretation and hypothesis generation. For the results and biological interpretation, we considered annotations on MSI level 3 (putatively characterised compound classes) [51].

MS2-based spectral similarity annotation and lipid search in MZmine provided the following hit counts: C18, 190 (negative mode) and 183 (positive mode), corresponding to approximately 6% of the detected features; C8, 99 (positive mode) representing 4% of the features. The annotation rates were significantly lower for C8 in negative mode and HILIC (both polarities), representing around 1% of the total number of features (Fig. 2B). The use of FBMN in GNPS2 enhanced MS2-based annotation using GNPS libraries and a modified cosine score for analogue search [45, 46]. Annotation rates were higher in C18 datasets, with 10% of the features annotated in both modes. In C8 datasets, 8% of features were annotated in positive mode, and 0.4% in negative mode. For HILIC, 4% in positive mode and 0.3% in negative mode. MS1-based annotation against a custom pollen compounds library resulted in a range of 20–240 hits (1–5% of total features) (Fig. 2B).

After combining the library-based annotations and removing duplicates, the AI-assisted review aided classification and exclusion of unlikely annotations, e.g., synthetic compounds, artefacts, and microbial natural products. The highest proportion of reliably annotated features was retained in C18 datasets (14–18%), followed by C8 (1–8%) and HILIC (2–5%). Annotation lists are available at Zenodo. SIRIUS MS2-based analyses using the CANOPUS chemical class prediction module assigned 14–60% (114 to 1,900 features), depending on the extraction and chromatographic column used (Fig. 2B). CANOPUS results were used to generate an overview of the metabolic profile obtained in each extraction and chromatographic method (Figure S5).

### Statistical analysis

**Fig. 3:**
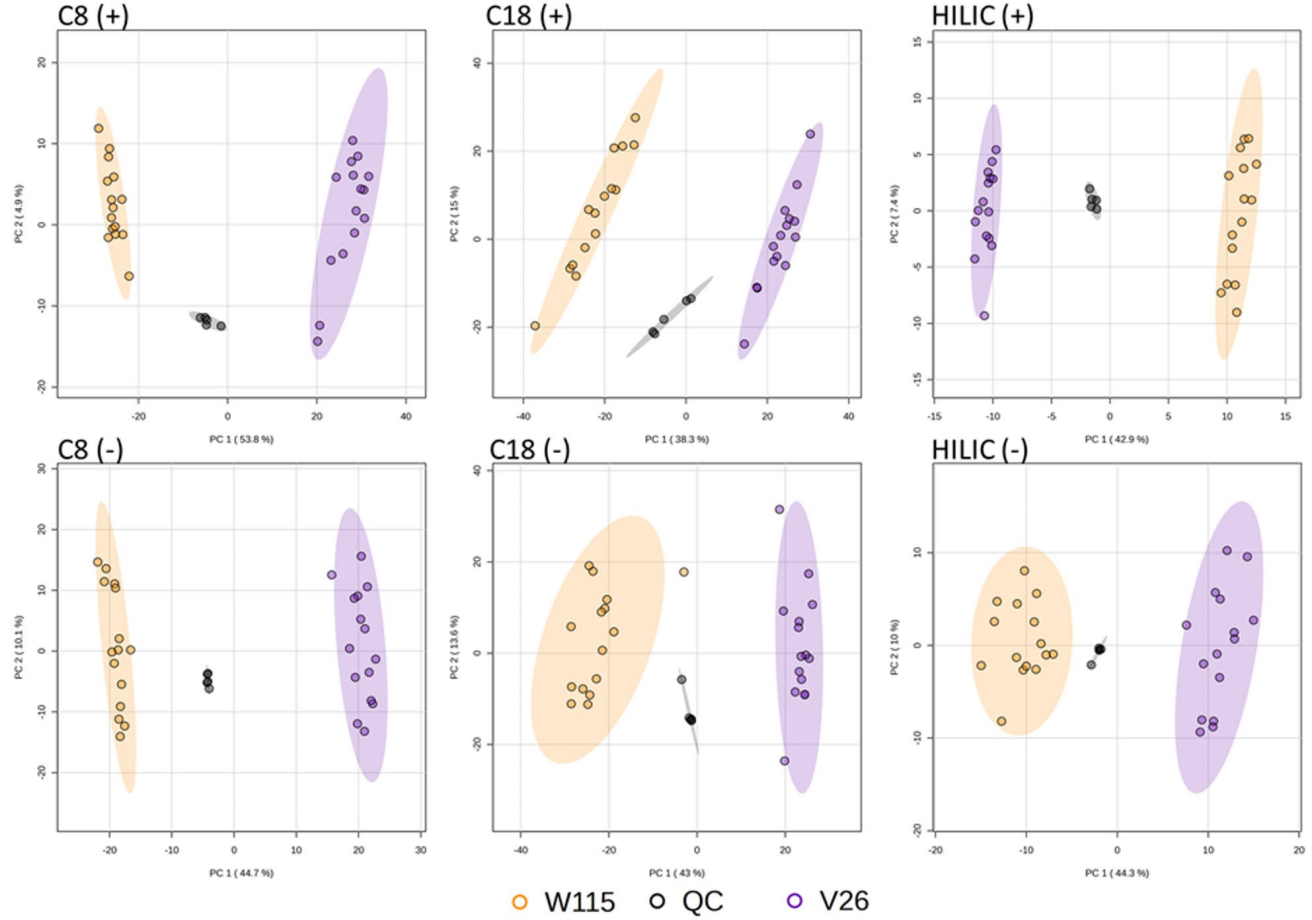
Principal Component Analysis 2D plots for datasets from *Petunia hybrida* pollen extracts. Data analysed using UHPLC-HRMS/MS using three different chromatographic columns (C8, C18, and HILIC) and two ionisation modes: (+) positive; (-) negative; W115 with white flowers: yellow; V26 violet flowers: violet dots; QC: quality control samples (grey dots). The coloured ovals represent the 95% intervals

Unsupervised multivariate analysis (PCA) revealed strong differences in the chemical profile of the pollen from the *P. hybrida* V26 and W115, primarily along with the first principal component (PC1) with 38% to 54% of explained variance across the different datasets (Fig. 3). Pooled-QC samples (grey dots, Fig. 3) grouped near the centre of the components, indicating appropriate data acquisition and processing [52]. Permutational Multivariate Analysis of Variance (PERMANOVA) confirmed the statistical significance of group separation (F-value > 50, R-squared > 0.75, and p-value = 0.001 based on 999 permutations - Table S10). As PCA evidenced a remarkable group separation, a rigorous statistical criterion (FDR-corrected p-value ≤ 1 × 10^− 5^) was used. This analysis revealed a high number of significant features differing among the datasets, ranging from 210 in the HILIC positive mode to 1,174 features in C18 negative mode (Table S10). The results were visualised using Volcano plots, showcasing and confirming a consistent difference in the chemical profile between the W115 and V26 groups (Figure S6). The hierarchical clustering heatmap of Pearson correlations for flavonoid and phenolamides identified by CANOPUS, revealed a negative correlation between the C18 datasets (Figure S7).

### Comparative chemical profiling

**Fig. 4:**
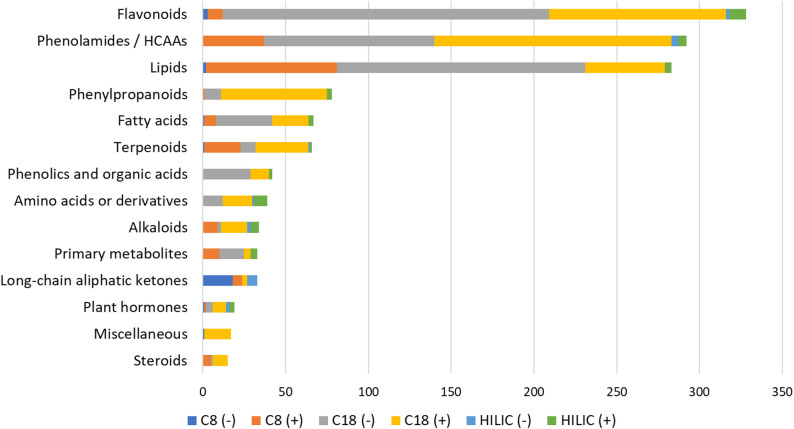
Numbers of features (*m*/*z*) assigned to different secondary metabolite classes annotated in *Petunia hybrida* pollen extracts from MS1 and MS2 libraries. Data analysed using UHPLC-HRMS/MS using three different chromatographic columns (C8, C18, and HILIC) and two ionisation modes: (+) positive; (-) negative

The main chemical classes annotated using MS1 and MS2 libraries were flavonoids (including glycosylated derivatives of quercetin, kaempferol, naringenin, and eriodyctiol), phenolamides (including mainly coumaroyl and feruloyl amides of spermidine), and lipids (including glycerophospholipids). Phenylpropanoids, fatty acids, amino acids, terpenoids, alkaloids were also annotated at chemical class level. Figure 4 presents an overview of the known chemical space of *P. hybrida* pollen based on their respective chemical classes and number of annotations from chemical and spectral libraries.

**Fig. 5:**
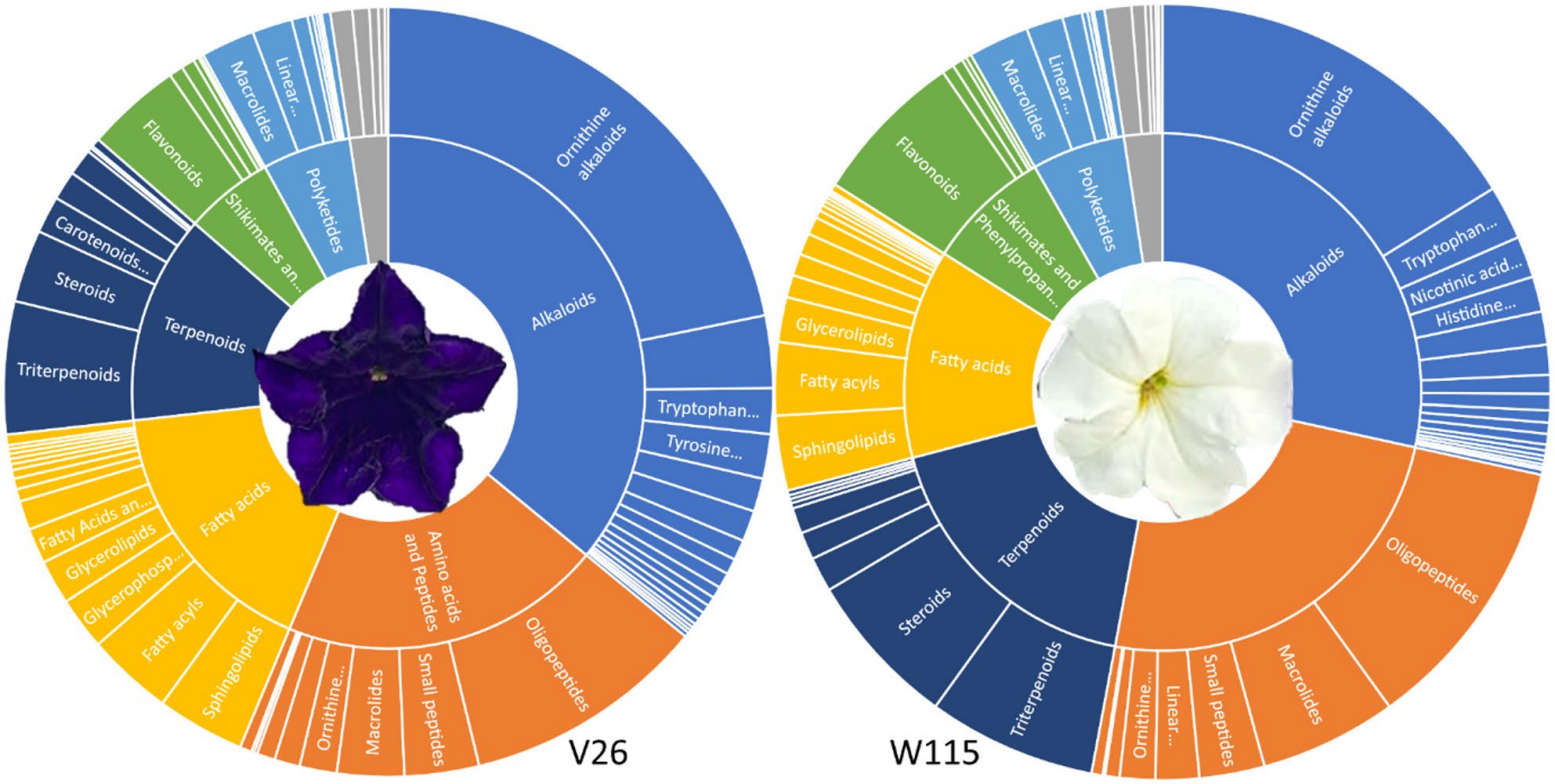
Combined sunburst plot of CANOPUS predicted compound classes in *Petunia hybrida* pollen extracts of two lines, V26 and W115, with differently coloured flowers. Data analysed using UHPLC-HRMS/MS using three different chromatographic columns (C8, C18, and HILIC) and two ionisation modes: (+) positive; (-) negative. Chemical classes structured hierarchically in accordance with the NPClassifier ontology. Individual slices are sized to the summed peak area of all classified features and arranged clockwise in descending order

We implemented the predicted annotations from CANOPUS to compare the metabolic profiles of pollen from both *Petunia* lines using metabolic pathways and superclasses based on NPClassifier chemical ontology [53] (Fig. 5). The main predicted chemical classes in *P. hybrida* pollen were polyamine alkaloids (ornithine-derived alkaloids, i.e., phenolamides), amino acids and peptides, glycerophospholipids, fatty acyls, sphingolipids, terpenoids (mainly triterpenoids and steroids), and flavonoids. Notably, pollen extracts from V26 contained more phenolamides (assigned as ornithine alkaloids by NPClassifier) and fatty acids, while flavonoids and terpenoids are more abundant in the pollen extract of W115. Figure 5 summarises the predicted chemical profile of *P. hybrida* pollen based on the summed peak area of all classified features across datasets, extending the known chemical space covered by chemical and spectral libraries. It highlights the main differences in the chemical profile of the pollen from each line. Individual chemical profiles dataset profiles are shown in Figure S8.

**Fig. 6:**
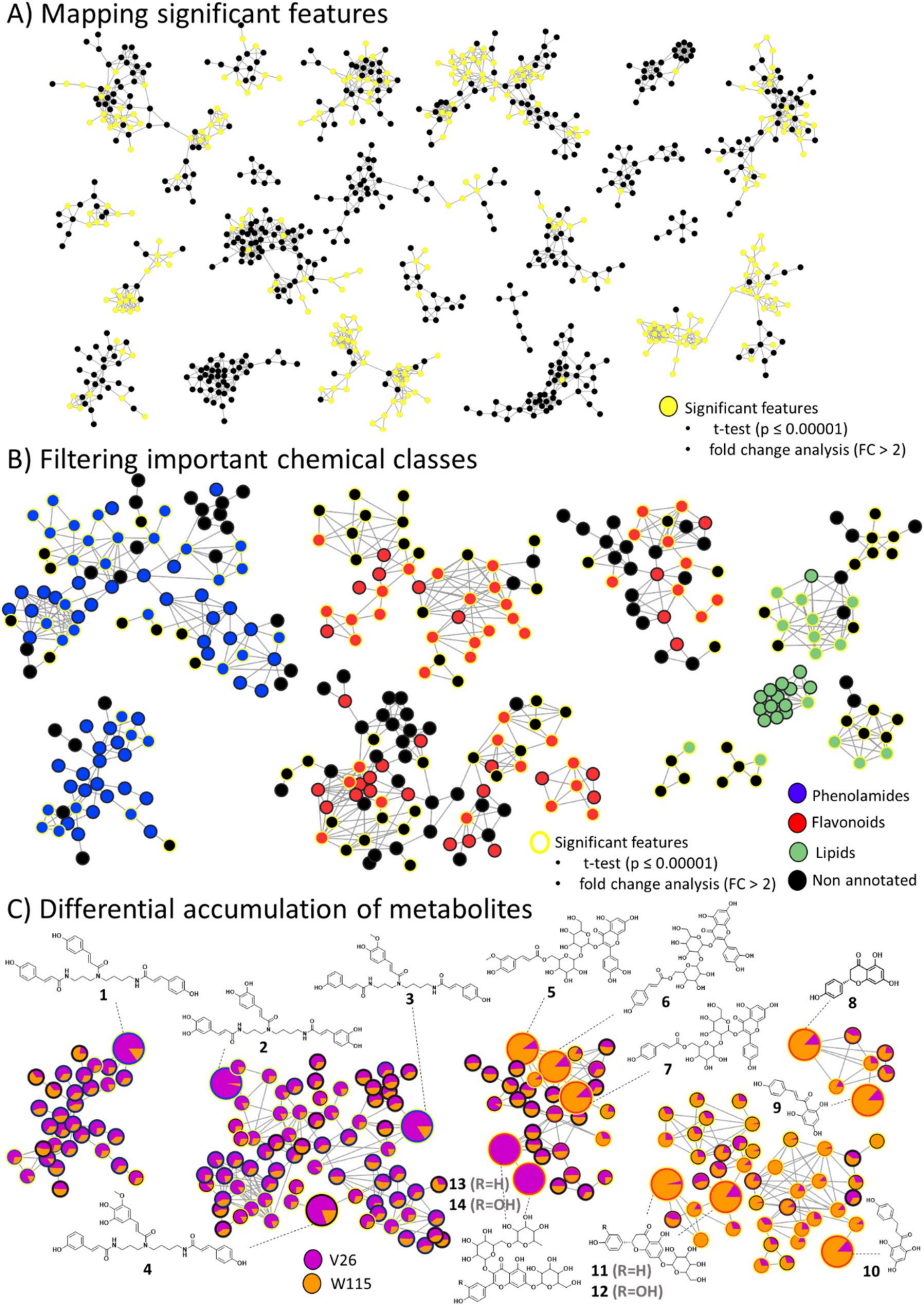
Integrated analysis of *Petunia hybrida* pollen extracts based on MS2 Feature-Based Molecular Networks (FBMNs) from GNPS 2 using Cytoscape. **A** Significant features from the Volcano plot (FDR-corrected *p* ≤ 1 × 10^− 5^ and FC > 2) highlighted as yellow nodes in the FBMNs. **B** Main annotated chemical classes in *P. hybrida* pollen mapped into FBMNs. **C** Distribution of features in pollen from lines V26 and W115 as pie chart nodes, highlighting annotations: (1) Tricoumaroyl-spermidine; (2) Tricaffeoyl-spermidine; (3) Dicoumaroyl-feruloyl-spermidine; (4) Dicoumaroyl-hydroxyferuloyl spermidine; (5) Quercetin-*O*-feruloyl-hexosyl-hexose; (6) Quercetin-*O*-coumaroyl-hexosyl-hexose; (7) Kaempferol-*O*-coumaroyl-hexosyl-hexose; (8) Naringenin; (9) Naringenin chalcone; (10) Phloretin (Dihydronaringenin); 11) Naringenin-*O*-hexose; 12) Eriodictyol-*O*-hexose; 13) Kaempferol-*O*-hexosyl-deoxyhexose-*O*-hexose; 14) Quercetin-*O*-hexosyl-deoxyhexose-*O*-hexose

Data integration further highlighted the differences between the metabolic patterns in the pollen of the two *Petunia*: most of the statistically significantly annotated flavonoids were more abundant in W115 pollen, whereas most of the annotated phenolamides and lipids were more abundant in V26 pollen (Figure S9). We mapped the statistically significant features from the Volcano plots (Figure S6), using thresholds of FDR-corrected p-values ≤ 1 × 10^− 5^ and a fold change > 2, onto the FBMNs (Fig. 6A) and their respective annotations (Fig. 6B) to highlight key chemical classes in *P. hybrida* pollen extracts. The analyses revealed statistically significant molecular families of phenolamides, flavonoid, and lipid derivatives (Fig. 6C).

## Discussion

### Pollen metabolic profiling

To our knowledge, this study provides the first comprehensive report of the chemical composition of *Petunia hybrida* V26 and W115 pollen. We used an untargeted metabolomics workflow tailored for *Petunia*, considering its flower morphology and pollen chemical complexity. Although wet pollen isolation methods (solvent-based) can cause rehydration of the pollen grains, previous studies indicated no significant effect on the metabolic profile of tomato pollen [32]. The 3-in-1 extraction method followed by fractionation allowed us to recover a wide range of metabolites, which is suitable when analysing complex matrices with limited quantities [54, 55]. The integration of complementary chromatographic methods with dual ionisation mass spectrometry enabled a comprehensive chemical characterisation.

The higher feature counts in hydromethanolic and organic extract phases analysed in reversed phase (C18 and C8 columns) highlighted the dominance of low- and medium-polarity metabolites in the pollen extracts, consistent with the non-polar nature of pollen [4]. While our results represent a metabolic snapshot of *P. hybrida* pollen under specific conditions, the use of complementary analytical platforms is necessary for maximising metabolome coverage, improve the annotation of isomeric species and properly report compounds instead of putative chemical compounds [56]. Non-polar and semi-polar specific methods, such as those utilising C8 and C18 chromatographic columns, also yielded the higher number of annotations for *P. hybrida* pollen. Among the main annotated compound classes (phenolamides, flavonoids, and lipids), we observed an interesting pattern of metabolite accumulation: phenolamides in V26, and flavonoids in W115 (Figures S7-S9), which is discussed in more details in the following sections.

By combining MS2 and MS1 spectral libraries with chemical class prediction algorithms, we aimed to mitigate the annotation bottleneck in untargeted metabolomics [57, 58]. However, the large number of detected features, the limited metabolite coverage in public repositories, and the vast and complex still unexplored “dark metabolome”, remain big challenges [59]. In-source fragmentation [60] and artifact ion formation [61] further complicate metabolite annotation. In addition, database-driven annotations are inherently biased by database content, which is often not taxa-specific, contains predicted spectra, and experimental data acquired under different analytical conditions [62]. Therefore, we built a curated and specific *in-house* library for compounds previously identified in pollen, but novel and species-specific compounds are likely to remain unannotated.

### Phenolamide and flavonoid in pollen from flowers with differing colours

Phenolamides are formed by a covalent linkage of phenolic hydroxycinnamic acid (HCA) moieties to aryl monoamines or aliphatic polyamines derived from L-arginine, such as spermidine, spermine, and putrescine [63]. Studies have highlighted their role in plant development and defence against biotic and abiotic stressors [64–66]. The presence of phenolamides in reproductive tissues is hypothesised to play an important role in protecting genetic information from UV radiation, in redox homeostasis, and in chemical interactions between plants, microbes and pollinators [67]. To date, there are 43 *N*-hydroxycinnamoyltransferase enzymes isolated and characterised that give place to a large diversity of chemical structures with different functions in plants [68]. Most of the annotated phenolamides in this work include di- and tri-substituted derivatives of spermidine with coumaroyl, caffeoyl, and feruloyl moieties (Table S11). The polyamine core and substituents were annotated using patterns of fragmentation and neutral losses, but the exact structural characterisation is complicated by the presence of positional isomers and stereoisomers [69]. Our findings evidenced dicoumaroyl-caffeoyl-spermidine, tricoumaroyl-spermine, tricaffeoyl-spermidine, dicoumaroyl-feruloyl- and dicoumaroyl-hydroxyferuloyl-spermidine as the main phenolamides accumulated in the mature pollen from V26, with violet flowers and bee-pollinated [21].

Flavonoids are characterised by a distinct 15-carbon skeleton arranged in two phenyl and one heterocyclic ring originated from building blocks from both phenylpropanoid (C6-C3) and acetate (3 x C2) pathways. Important subclasses include flavones, flavonols, flavanones, and anthocyanidins [70]. Flavonoids in pollen are important for germination and successful reproduction, protection against UV irradiation, and as antioxidants [71]. Importantly, levels of flavonoids in flowers have been linked to pollinator preferences in *Petunia* plants [72]. Flavanone and flavonol derivatives were the major subclasses annotated in *Petunia* pollen; a finding consistent with reports that have described naringenin in the sporopollenin wall, while kaempferol and quercetin have been found in the protoplasm [73]. The W115 pollen extract showed accumulation of glycosylated flavanones naringenin and eriodyctiol (Table S11). Glycosylated flavonols quercetin and kaempferol conjugated with HCA (*O*-coumaroyl and *O*-feruloyl di-glycosides) were also accumulated in the pollen from the W115 line, while simple glycosylated derivatives (mainly *O*-di-glycosides) of flavonols kaempferol, quercetin and rhamnetin were present in higher abundance in pollen extract from the V26 (Table S11). Flavonol 3-*O*-di-glycosides often accumulate as major flavonoids in pollen, and kaempferol 3-*O*-glucosyl-(1→2)-galactoside have already been described in the pollen of *P. hybrida* V26 [30, 74].

**Fig. 7:**
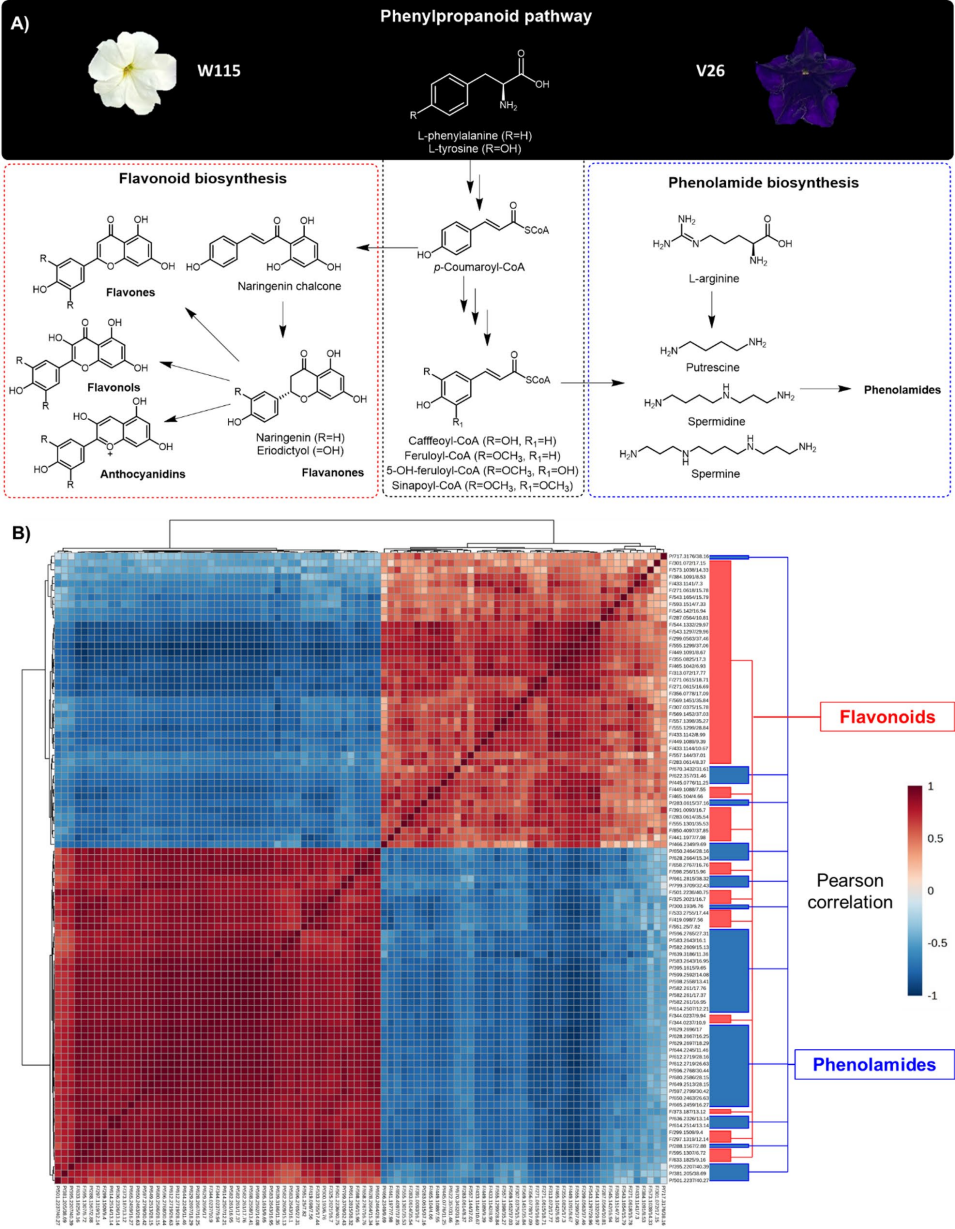
Flavonoid-phenolamide dichotomy in *Petunia hybrida* pollen (W115 and V26). **A** Schematic representation of the biosynthetic divergence between flavonoid and phenolamide classes from the phenylpropanoid metabolism. **B** Hierarchically clustered heatmap of Pearson correlation coefficients among CANOPUS-annotated flavonoid and phenolamides features detected in *P. hybrida* pollen extracts by UHPLC-HRMS/MS (C18, negative mode). Features are labelled as ‘F’ (flavonoids, red brackets) and ‘P’ (phenolamides, blue brackets), followed by *m/z* and retention time. Clustering highlights two major co-varying modules associated with W115 (red) and V26 (blue)

The accumulation of flavonoid glycosides conjugated with HCA in W115 pollen aligns with a higher prevalence of phenolamides in the V26 pollen, as both compound classes share, in part, a common biosynthetic origin. These results suggest a potential metabolic trade-off between these secondary metabolite pathways in pollen of *Petunia* with different flower colours (Fig. 7). Despite the cellular compartmentalisation of flowers and anthers, their primary and secondary metabolism is connected and coordinated [75]. Our findings in *Petunia* pollen might be correlated to the flavonoid metabolism in its flowers: active biosynthesis of anthocyanins in violet flowers leads to phenylpropanoid pathway directed to phenolamides in pollen, while the lack of anthocyanin biosynthesis in white flowers leads to a higher presence of flavonoid derivatives in pollen. Further research is needed to fully corroborate this hypothesis, for instance, flux analyses including quantification of relevant intermediates in floral tissues, and analyses on the expression of key biosynthetic enzymes such as 4-coumarate: CoA ligase (4CL), hydroxycinnamoyl-CoA: shikimate hydroxycinnamoyl transferase (HCT), and phenylpropanoid transporters.

The phenylpropanoid metabolism is highly conserved in the pollen of angiosperms [76], but the function of secondary metabolites derived from this pathway in pollen is not completely clear. The observed trade-off between flavonoids and phenolamides in the pollen of *P. hybrida* W115 and V26 suggests a specific regulation of the phenylpropanoid pathway between these lines [77]. The flavonoid/phenolamide balance in Solanaceae has been associated with mechanisms of adaptation [78–80], and given the secondary metabolism in plants highly conditioned by environmental pressures [81], we hypothesise that the evolutionary relationships of *Petunia* are likely shaped also by metabolic differences in their pollen.

### Pollen secondary metabolism and pollination

Pollinator preference in *Petunia* is mainly attributed to floral traits, such as petal colour, scent, and morphology [82]. The speciation of white-flowered *P. axillaris* from purple-flowered *P. inflata*-like ancestors involved the loss of anthocyanin floral pigments in exchange for floral scents, and a shift from bee to moth pollination [21]. Our findings suggest that differences in pollen chemistry, more specifically flavonoids and phenolamides, might be an additional cue for pollinators.

The overall prevalence of flavonoids observed in pollen of W115 line aligns with findings of accumulation of these compounds in UV-absorbing white flowers of the ancestral *P. axillaris* (moth-pollinated) in comparison with the lower levels found in *P. inflata* (violet and bee-pollinated flowers) and *P. exserta* (red and hummingbird-pollinated flowers) [72]. It has been suggested that high levels of flavonols in *P. axillaris* evolved to attract nocturnal visitors, such as hawkmoths [83]. Furthermore, phenolamides are important for chemical defence of wild tobacco (*Nicotiana attenuata*) against *Manduca sexta* [84], which is the main pollinator of the white flower *P. axillaris* [83]. Our findings of lower levels of phenolamides and higher flavonoid levels in W115 pollen, contrasted with phenolamide accumulation in V26 pollen, suggest their possible role in plant-pollinator interaction.

Mutualistic interactions also support the accumulation of secondary metabolites in pollen – plants secure the reproduction in exchange for bioactive compounds for pollinator benefit. Evidence of the use of pollen secondary metabolites by pollinators has been demonstrated in stingless bee-plant symbiosis; both plant flavonoids and phenolamides were detected across different beehive structures, bee developmental stages, and bee products [85, 86]. These compounds seem to be essential components for establishing a healthy microbiota in *Tetragonisca fiebrigi* honey and beehives [85], and are also the most diverse natural products identified in the *Scaptotrigona depilis* – plant symbiosis, being present in bee-pollen, honey, and propolis [86]. In addition, the diferuloyl-spermidine and coumaroyl-feruloyl-spermidine were detected in pollen, larvae, brood fungus, larvae, cerumen, and entrance of the colony of *S. depilis* [86]. Thus, phenolamides and flavonoids may present important functional roles for bees and moths, which can also support their choice for visiting specific *Petunia* flower morphs.

### Conclusions and future perspectives

We effectively characterised the chemical space of mature pollen from *P. hybrida* cultivar V26 and W115 using untargeted metabolomics, suggesting a trade-off between phenolamides and flavonoids. Our results contribute to understanding the role of secondary metabolites in *Petunia* pollen and hypothesise its plausible ecological implications; while floral traits are known drivers of pollination syndromes [87, 88], pollen secondary metabolites may also participate [5]. Future research should focus on elucidating the chemical differences in wild parents (*P. axillaris* and *P. inflata*) and the specific effects of phenolamides and flavonoids in pollinator behaviour. Integration of different omics data, e.g., additional metabolic flux and metabolomic analyses to identify key intermediates in other floral tissues (anthers and petals), combined with transcriptomic and proteomic measurements of branch point enzyme production to directly test pathway competition, would be desirable for better understanding of pollen metabolic dynamics. The pollen analysis workflow for *Petunia* plants, the open-access custom pollen MS1 library, and our publicly available metabolomic data [89] are valuable resources for advancing pollen metabolomics. Furthermore, this study highlights the value of UHPLC-HRMS/MS combined with open-source and cutting-edge bioinformatic tools for chemical profiling underexplored biological matrices.

## Supplementary Information

Supplementary Material 1.

## Data Availability

The dataset generated and analysed during the current study are available at Zenodo (10.5281/zenodo.18684813). Supplementary tables, figures, and texts supporting the conclusions of this article are included within the article and its additional files.

## Abbreviations

Abbreviation: Definition
4CL: 4-coumarate: CoA ligase
AI: Artificial intelligence
CID: Collision-induced dissociation
CSV: Comma separate values
DDA: Data-dependent acquisition
ESI: Electrospray ionisation
FBMN: Feature-based molecular networking
FC: Fold change
FDR: False discovery ratio
GABA: Gamma-amino butyric acid
GNPS: Global natural product social molecular networking
HCA: Hydroxycinnamic acid
HCAA: Hydroxycinnamic acid amides
HCT: Hydroxycinnamoyl-CoA: shikimate hydroxycinnamoyl transferase
HILIC: Hydrophilic interaction liquid chromatography
HRMS: High resolution mass spectrometry
ID: Identification number
IMS: Ion mobility spectrometry
LOD: Limit of detection
MGF: Mascot generic format
MSI: Metabolomics standards initiative
MW: Molecular weight
m/z: Mass-to-charge ratio
PCA: Principal component analysis
PERMANOVA: Permutational multivariate analysis of variance
QC: Quality control
QTOF: Quadrupole time-of-flight
Rcf: Relative centrifugal force
RT: Retention time
UHPLC-HRMS/MS: Ultra-high-performance liquid chromatography coupled to high resolution in tandem mass spectrometry
UV: Ultra-violet radiation
