# Supplementary material for: Untargeted metabolomics suggests a trade-off between phenolamides and flavonoids in the pollen of Petunia hybrida with contrasting flower colours
Source: BMC Plant Biol. 2026 Jun 11;26:1410. doi: 10.1186/s12870-026-08968-y (PMC13483416; doi:10.1186/s12870-026-08968-y)
Supplement: Supplementary file 1 [file 12870_2026_8968_MOESM1_ESM.docx]

**Supplementary Material**

**Untargeted metabolomics suggests a trade-off between phenolamides and flavonoids in the pollen of *Petunia hybrida* with contrasting flower colours**

Matheus Fernandes Alves^1,2†^, Abigail Moreno-Pedraza^1,2,3†*^, Paula Carolina Pires Bueno^2^, Khabat Vahabi^2^ and Nicole M. van Dam^1,2*^

*^1^Institute of Biodiversity, Ecology, and Evolution (IBEE), Friedrich Schiller University Jena, Dornburgerstraße 159, 07743 Jena, Germany*

*^2^Leibniz Institute of Vegetable and Ornamental Crops (IGZ), Theodor-Echtermeyer-Weg 1, 14979 Großbeeren, Germany*

*^3^Earth and Biological Sciences Directorate, Pacific Northwest National Laboratory, Richland, Washington 99354, United States*

† These authors contributed equally to this work

^*^Correspondent authors e-email addresses*:* [*abigail.moreno@pnnl.gov*](mailto:abigail.moreno@pnnl.gov) and [*vandam@igzev.de*](mailto:vandam@igzev.de)

**Content:**

**Supplementary tables**………………………………………………………………….….…….…...2

**Supplementary images**…………………………………………………………………….……….34

**Supplementary text**……………………………………………………………….………….…….43

**Supplementary tables**

**Table S1. Chromatographic parameters employed for UHPLC-HRMS/MS data acquisition.**

| **Chromatographic column** | **Eluent A** | **Eluent B** | **Gradient, volume of injection, flow rate, and oven temperature** |
| --- | --- | --- | --- |
| C8  (Eclipse®) | MilliQ H_2_O  + 0.1% FA | ACN + 0.1% FA | **Gradient**: 10% of B from 0 to 1 min, then from 10 to 40% of B from 1 to 2 min, keeping at 40% of B from 2 to 3 min, then from 40 to 60% of B from 3 to 14 min, then from 60 to 98% of B from 14 to 23 min, keeping at 98% from 23 to 26 min, returning to 10% of B from 26.0 to 26.1 min and keeping at 10% of B until 33 min to reconditioning.  **Volume of injection**: 3 µL, **flow rate** 0.4 mL/min. **Oven temperature**: 40 °C. |
| C18  (Poreshell 120®) | MilliQ H_2_O  + 0.1% FA | ACN + 0.1% FA | **Gradient**: 5% of B from 0 to 1 min, then from 5 to 20% of B from 1 to 6 min, then from 20 to 25% of B from 6 to 12 min, keeping at 25% of B from 12 to 20 min, then from 25 to 30% of B from 20 to 28 min, from 30 to 95% of B from 28 to 48 min, keeping at 95% of B from 28 to 51 min, returning to 5% of B from 51 to 52 min and keeping at 5% of B until 57 min to reconditioning.  **Volume of injection**: 3 µL, **flow rate** 0.4 mL/min. **Oven temperature**: 40 °C. |
| HILIC  (HILIC-Z®) | Aqueous NH_4_HCO_2_ 1mM buffer + 0.1% FA | ACN/H_2_O 9:1 (*v*/*v*)  + 0.1% FA | **Gradient**: 90% of B from 0 to 5 min, then from 90 to 30% of B from 5 to 9 min, keeping at 30% of B from 9 to 10 min, returning to 90% of B from 10.0 to 10.1 min and keeping at 90% of B until 13 min to reconditioning.  **Volume of injection**: 1 µL, **flow rate** 0.4 mL/min. **Oven temperature**: 30 °C. |

**Table S2. List of software and platforms employed for data processing.**

| **Name** | **Version/release** | **Repository/download** | **Documentation** |
| --- | --- | --- | --- |
| MSConvert | 3.0.24213 | <https://proteowizard.sourceforge.io/download.html> | <http://proteowizard.sourceforge.io/doc_users.html> |
| MZmine | 4.7.29 | <https://github.com/mzmine/mzmine/releases/tag/v4.7.29> | <https://mzmine.github.io/mzmine_documentation/index.html> |
| Sirius | 6.2.0 | <https://github.com/sirius-ms/sirius> | <https://v6.docs.sirius-ms.io/> |
| MetaboAnalyst | 6.0 | <https://www.metaboanalyst.ca/> | <https://metaboanalyst.ca/docs/Tutorials.xhtml> |
| GNPS 2 | 2025.06.17 | <https://gnps2.org/homepage> | <https://ccms-ucsd.github.io/GNPSDocumentation/> |
| Cytoscape | 3.10.3 | <https://cytoscape.org/> | <https://github.com/cytoscape/cytoscape-tutorials/wiki> |

**Table S3. Parameters set for data conversion using MSConvert from Proteowizard (3.0.24213).**

| **Parameter** | **Set** |
| --- | --- |
| Binary Encoding Precision | 64-bit |
| Compression Method | Zlib |
| TPP Compatibility | Enabled |
| Index Write | Enabled |
| Filter | Peak Picking |
| Algorithm/ MS Levels | Vendor/ 1 and 2 |

**Table S4. Parameters used for feature extraction in MZmine (4.7.29).**

| **Module (parameter)** | **Set** | | |
| --- | --- | --- | --- |
| **Method** | **C8** | **C18** | **HILIC** |
| Mass detection  (MS1 noise level) | 2E3 | 2E3 | 4E3 (+) / 2E3 (-) |
| Mass detection  (MS2 noise level) | 8E2 (+) / 4E2 (-) | 8E2 | 7E2 (+) / 4E2 (-) |
| Chromatogram builder (minimum scans) | 3 | 3 | 2 |
| Chromatogram builder (minimum intensity for consecutive scans) | 8E3 (+) / 5E3 (-) | 4E3 | 6E3 |
| Chromatogram builder (minimum absolute height) | 5E4 (+) / 1E4 (-) | 2E4 (+) / 1E4 (-) | 1E4 |
| Chromatogram builder  (*m/z* tolerance) | 0.007 *m/z* or 12 ppm | 0.012 *m/z* or 15 ppm (+) / 0.025 *m/z* or 15 ppm (-) | 0.012 *m/z* or 15 ppm |
| Smoothing - Savitzky Golay  (RT width in scans) | 7 | 5 | 5 |
| Local minimum  feature resolver  (MS/MS pairing using feature edges) | 0.01 *m/z* or 12 ppm, 25% | 0.02 *m/z* or 15 ppm, 25% | 0.02 *m/z* or 15 ppm, 25% |
| Local minimum  feature resolver  (RT dimension) | 85% threshold, search range of 0.05 min | 85% threshold, search range of 0.04 min (+) / 0.05 min (-) | 85% threshold, search range of 0.05 min |
| Local minimum  feature resolver  (absolute height, peak top/edge, and scans) | 1E5 (+) / 5E4 (-), 1.70, and 3 | 2E4 (+) / 1E4 (-), 1.70, and 2 | 1E4, 1.7, and 2 |
| ^13^C isotope resolver  (*m/z* tolerance) | 0.005 *m/z* or 10 ppm | | |
| ^13^C isotope resolver  (RT tolerance) | 0.05 min | | |
| Join aligner  (*m/z* tolerance, weight 3) | 0.015 *m/z* or 15 ppm (+) / 0.02 *m/z* or 20 ppm (-) | 0.025 *m/z* or 20 ppm (+) / 0.05 *m/z* or 20 ppm (-) | 0.045 *m/z* or 20 ppm (+) / 0.25 *m/z* or 20 ppm (-) |
| Join aligner  (RT tolerance, weight 3) | 0.1 min (+) / 0.15 min (-) | 0.35 min (+) / 0.5 min (-) | 0.1 min (+) / 0.2 min (-) |
| Feature finder  (*m/z* tolerance) | 0.005 *m/z* or 7 ppm (+) /  0.01 *m/z* or 15 ppm (-) | 0.02 *m/z* or 15 ppm (+) / 0.035 *m/z* or 15 ppm (-) | 0.035 *m/z* or 15 ppm (+) / 0.01 *m/z* or 15 ppm (-) |
| Feature finder  (RT tolerance) | 0.07 min (+) / 0.1 min (-) | 0.3 min | 0.12 min |
| Feature finder  (minimum scans) | 2 | | |
| Duplicate peak filter  (new average) | 0.005 *m/z* or 7 ppm (+) / 0.03 *m/z* or 20 ppm (-) | 0.05 *m/z* or 20 ppm | 0.06 *m/z* or 20 ppm (+) / 0.02 or 20 ppm (-) |
| Duplicate peak filter  (new average) | 0.06 min | 0.25 min (+) / 0.35 min (-) | 0.15 min |
| Feature list rows filter  (RSD filter using QC samples, peak area, and LOD 1/5 of global minimum imputation) | Maximum missing values 30%, Maximum RSD 30% | | |
| Correlation grouping  (feature shape and  height correlation) | RT tolerance of 0.05 min | | |
| Ion Identity networking  (all features) | 0.007 *m/z* or 10 ppm tolerance, 0 min height, all adducts | | |
| Feature list  blank subtraction  (quantification using area and average ratio type) | Minimum detection in 1 blank, fold change increase of 500% | | |

**Table S5. Parameters used in the spectral search module in MZmine for MS2 annotation.**

| **Parameter** | **Value** |
| --- | --- |
| Mass Tolerance | 0.01 *m*/*z* or 15 ppm |
| Precursor Tolerance | 0.008 *m*/*z* or 7 ppm |
| Spectral Tolerance (Fragments) | 0.01 *m*/*z* or 15 ppm |
| Minimum Matched Fragments | 4 |
| Precursor Signal | Keep |
| Similarity Algorithm | Weighted cosine with MassBank weight |
| Minimum Cosine Similarity | 0.7 |

**Table S6. Parameter used in the local compound database search module in MZmine for MS1 annotation.**

| **Parameter** | **Value** |
| --- | --- |
| Mass tolerance | 0.01 *m*/*z* or 5 ppm |
| Adducts in positive mode | [M+H]^+^ |
| Adducts in negative mode | [M-H]^-^ |
| Maximum charge | 2 |
| Maximum molecules per cluster | 2 |

**Table S7. Parameters used for FBMN in GNPS 2.**

| **Parameter** | **Value** |
| --- | --- |
| Precursor Ion Tolerance | 0.01 Da |
| Fragment Ion Tolerance | 0.02 Da |
| Filters Applied | Window and precursor window filters |
| Minimum Cosine Similarity  (networking and library) | 0.7 |
| Minimum Matched Peaks  (networking and library) | 6 |
| Top K | 10 |
| Maximum Component Size  Analog search  Analog search maximum shift | 100  Yes  500 |

**Table S8. GNPS Feature-based molecular networking task links.**

| **Dataset** | **Task ID** | **Access link** |
| --- | --- | --- |
| C8 (ESI-) | f98b1b6978574dedb483c8b3ccb7389f | <https://gnps2.org/status?task=9b531c3913ed496691ff329138a864ff> |
| C8 (ESI+) | 9b531c3913ed496691ff329138a864ff | <https://gnps2.org/status?task=f98b1b6978574dedb483c8b3ccb7389f> |
| C18 (ESI-) | e79456d1342f443bad85b8b855f66acc | <https://gnps2.org/status?task=e79456d1342f443bad85b8b855f66acc> |
| C18 (ESI+) | a16c9467f1694ea39c2324d3c1c52356 | <https://gnps2.org/status?task=a16c9467f1694ea39c2324d3c1c52356> |
| HILIC (ESI-) | ac181ef8241d488bbbb0f6b3b9d2c24c | <https://gnps2.org/status?task=ac181ef8241d488bbbb0f6b3b9d2c24c> |
| HILIC (ESI+) | eb8fcb4417e7447b9a30d9b3fd9818f3 | <https://gnps2.org/status?task=eb8fcb4417e7447b9a30d9b3fd9818f3> |

**Table S9. Parameter set to SIRIUS pipeline for MS2-based predictions.**

| **Parameter** | **Value** |
| --- | --- |
| Instrument Type | Q-TOF |
| MS2 Mass Accuracy | 15 ppm |
| Adducts (Positive and negative mode) | [M+H]^+^ and [M-H]^-^, respectively |
| Databases Selected for Search | All |
| Analogue Spectral Library Search (Precursor Deviation) | 20 ppm |
| Molecular Formula Identification Strategy | “De novo + bottom up” (Perform de novo to *m*/*z* up to 2000) |
| Elements Filter for "De novo" | Hydrogen (H), Carbon (C), Nitrogen (N), Oxygen (O), Phosphorus (P), Sulfur (S) |
| Modules for Fingerprint Prediction | CSI:FingerID with score threshold |
| Compound Class Prediction Module | CANOPUS with score threshold |
| Structure Database Search | CSI:FingerID performed with PubChem as fallback (approximation in confidence mode) |
| De Novo Structure Generation Module | MSNovelist |

**Table S10. Results from PCA, t-test and Volcano plot from *Petunia hybrida* pollen extracts.** Samples analysed by UHPLC-HRMS/MS using three different chromatographic columns (C8, C18, and HILIC) and two ionisation modes: (+) positive; (-) negative.

|  | **Principal Component Analysis**  **(5 components)** | | | | **Student's**  **t-test (p>0.00001)** | **Volcano’s significant features in each group**  **(p>0.00001, FC 2)** | |
| --- | --- | --- | --- | --- | --- | --- | --- |
| **Dataset** | **Explained variance** | **F-value** | **R^2^** | **p-value** | **Significant features** | **W115** | **V26** |
| C8 (-) | 67.2 | 72.7 | 0.82 | 0.001 | 820 | 467 | 219 |
| C8 (+) | 69.1 | 266.1 | 0.94 | 0.001 | 1061 | 606 | 390 |
| C18 (-) | 73 | 55.6 | 0.78 | 0.001 | 1174 | 406 | 593 |
| C18 (+) | 68.1 | 54.0 | 0.77 | 0.001 | 921 | 370 | 445 |
| HILIC (-) | 70.6 | 59.7 | 0.79 | 0.001 | 309 | 108 | 155 |
| HILIC (+) | 65.0 | 90.6 | 0.85 | 0.001 | 210 | 63 | 124 |

**Table S11. Statistically significant compounds annotated in *Petunia hybrida* pollen extracts.**

| **C8 (+)** | | | | | | | | | |
| --- | --- | --- | --- | --- | --- | --- | --- | --- | --- |
| RT (min) | Observed*m/z* | Compound name | Molecular formula | Adduct | Error (ppm) | Chemical class | log_2_(FC) | MSI | Source |
| 4.08 | 660.2915 | Diferuloyl-caffeoyl-spermidine isomer | C_36_H_41_N_3_O_9_ | [M+H]^+^ | -0.1 | Phenolamide | -2.33 | 3 | Pollen library |
| 4.82 | 273.0759 | Naringenin | C_15_H_12_O_5_ | [M+H]^+^ | 0.5 | Flavonoid | -3.08 | 3 | Spectral match |
| 5.76 | 614.2856 | Dicoumaroyl-feruloyl-spermidine isomer | C_35_H_39_N_3_O_7_ | [M+H]^+^ | -0.8 | Phenolamide | 3.12 | 3 | Pollen library |
| 7.35 | 635.2917 | Rescinnamine-like alkaloid | C_35_H_42_N_2_O_9_ | [M+H]^+^ | -7.3 | Alkaloid | 1.65 | 3 | Spectral match |
| 10.07 | 600.2682 | Dicoumaroyl-caffeoyl-spermidine isomer | C_34_H_37_N_3_O_7_ | [M+H]^+^ | -3.7 | Phenolamide | -3.64 | 3 | Pollen library |
| 10.10 | 641.3408 | Tricoumaroyl-spermine isomer | C_37_H_44_N_4_O_6_ | [M+H]^+^ | 11.6 | Phenolamide | 4.80 | 3 | Pollen library |
| 10.59 | 635.3358 | Sungucine-like alkaloid | C_42_H_42_N_4_O_2_ | [M+H]^+^ | -3.5 | Alkaloid | -4.33 | 3 | Spectral match |
| 12.83 | 653.2537 | Iridoid-*O*-acylglycoside | C_31_H_40_O_15_ | [M+H]^+^ | 14.8 | Terpenoid | -6.43 | 3 | Pollen library |
| 14.70 | 298.2739 | Sphingosine (18:2) | C_18_H_35_NO_2_ | [M+H]^+^ | -0.5 | Lipid | 1.83 | 3 | Lipid module MZmine |
| 14.79 | 619.3419 | Fatty-acid derivative | C_30_H_50_O_13_ | [M+H]^+^ | 15.3 | Fatty Acid | -1.58 | 3 | Pollen library |
| 14.79 | 579.3183 | Dicoumaroyl-di-acetyl-spermine isomer | C_32_H_42_N_4_O_6_ | [M+H]^+^ | 1.0 | Phenolamide | -2.86 | 3 | Pollen library |
| 14.80 | 584.2730 | Tricoumaroyl-spermidine isomer | C_34_H_37_N_3_O_6_ | [M+H]^+^ | -4.3 | Phenolamide | -2.85 | 3 | Pollen library |
| 15.50 | 318.3002 | Phytosphingosine | C_18_H_39_NO_3_ | [M+H]^+^ | -0.2 | Lipid | -2.07 | 2 | Spectral match |
| 16.23 | 520.3398 | LPC 18:2 | C_26_H_50_NO_7_P | [M+H]^+^ | 0.1 | Lipid | 2.30 | 3 | Spectral match |
| 17.01 | 454.2926 | LPE 16:0 | C_21_H_44_NO_7_P | [M+H]^+^ | -0.5 | Lipid | 2.08 | 3 | Lipid module MZmine |
| 17.14 | 279.2319 | Linolenic acid | C_18_H_30_O_2_ | [M+H]^+^ | 0.2 | Fatty Acid | 1.59 | 2 | Spectral match |
| 23.96 | 589.4820 | SQDG (16:1/18:3) | C_37_H_64_O_5_ | [M+H]^+^ | -1.1 | Lipid | 1.37 | 3 | GNPS |
| **C18 (-)** | | | | | | | | | |
| 3.19 | 259.1303 | Gamma-Glutamylleucine | C_11_H_20_N_2_O_5_ | [M-H]^-^ | 1.4 | Amino acid or derivative | 1.54 | 2 | Spectral match |
| 4.33 | 771.1987 | Kaempferol-*O*-hexosyl-hexose-*O*-hexose | C_33_H_40_O_21_ | [M-H]^-^ | -0.3 | Flavonoid | -5.05 | 3 | Spectral match |
| 4.33 | 137.0245 | Protocatechuic aldehyde | C_7_H_6_O_3_ | [M-H]^-^ | 0.6 | Phenolic derivative | 1.33 | 2 | Spectral match |
| 4.40 | 355.1037 | Feruloyl hexoside | C_16_H_20_O_9_ | [M-H]^-^ | 0.7 | Phenylpropanoid Glycoside | -5.23 | 2 | Spectral match |
| 4.57 | 175.0614 | Isopropylmalic acid | C_7_H_12_O_5_ | [M-H]^-^ | 1.1 | Organic acid | -2.91 | 2 | Spectral match |
| 4.66 | 465.1040 | Dihydroquercetin-*O*-hexose isomer | C_21_H_22_O_12_ | [M-H]^-^ | 0.3 | Flavonoid | -3.74 | 3 | GNPS |
| 4.93 | 325.0931 | Coumaroyl hexoside | C_15_H_18_O_8_ | [M-H]^-^ | 0.6 | Phenylpropanoid glycoside | -2.50 | 2 | Spectral match |
| 4.99 | 449.1089 | Dihydrokaempferol*-O*-hexose | C_21_H_22_O_11_ | [M-H]^-^ | -0.1 | Flavonoid | -5.55 | 3 | Spectral match |
| 5.00 | 627.1560 | Kaempferol derivative | C_27_H_32_O_17_ | [M-H]^-^ | -1.1 | Flavonoid | 2.68 | 3 | GNPS |
| 5.06 | 465.1037 | Dihydroquercetin *O*-hexose isomer | C_21_H_22_O_12_ | [M-H]^-^ | -0.3 | Flavonoid | -2.33 | 3 | Spectral match |
| 5.09 | 278.0670 | *N*-coumaroyl-aspartate | C_13_H_13_NO_6_ | [M-H]^-^ | 0.0 | Amino acid or derivative | 1.73 | 2 | GNPS |
| 5.17 | 177.0195 | Esculetin | C_9_H_6_O_4_ | [M-H]^-^ | 0.9 | Coumarin | 1.39 | 2 | Spectral match |
| 5.59 | 371.0987 | 6-(3-benzoyloxy-2-hydroxypropoxy)-3,4,5-trihydroxyoxane-2-carboxylic acid isomer | C_16_H_20_O_10_ | [M-H]^-^ | 0.9 | Organic acid | -3.15 | 3 | Spectral match |
| 6.45 | 287.0563 | Dihydrokaempferol | C_15_H_12_O_6_ | [M-H]^-^ | 0.6 | Flavonoid | -3.95 | 2 | Spectral match |
| 6.47 | 371.0986 | 6-(3-benzoyloxy-2-hydroxypropoxy)-3,4,5-trihydroxyoxane-2-carboxylic acid isomer | C_16_H_20_O_10_ | [M-H]^-^ | 0.6 | Organic acid | -2.03 | 3 | Spectral match |
| 7.12 | 639.1566 | Rhamnetin-*O*-di-hexose | C_28_H_32_O_17_ | [M-H]^-^ | -0.1 | Flavonoid | 1.86 | 3 | Spectral match |
| 7.30 | 433.1141 | Naringenin *O*-hexose | C_21_H_22_O_10_ | [M-H]^-^ | 0.2 | Flavonoid | -1.59 | 3 | Spectral match |
| 7.33 | 801.1883 | Quercetin-*O*-feruloyl-hexosyl-hexose | C_37_H_38_O_20_ | [M-H]^-^ | -0.1 | Flavonoid | -3.22 | 3 | GNPS |
| 7.49 | 653.1722 | Rhamnetin derivative | C_29_H_34_O_17_ | [M-H]^-^ | -0.2 | Flavonoid | 1.84 | 3 | Manual |
| 8.18 | 755.1827 | Kaempferol-*O*-coumaroyl-hexosyl-hexose | C_36_H_36_O_18_ | [M-H]^-^ | -0.3 | Flavonoid | -2.88 | 3 | GNPS |
| 8.29 | 435.1285 | Phloretin-*O*-hexoside isomer | C_21_H_24_O_10_ | [M-H]^-^ | -2.7 | Flavonoid | -3.00 | 3 | Pollen library |
| 8.29 | 431.0985 | Genistein-*O*-hexoside | C_21_H_20_O_10_ | [M-H]^-^ | 0.3 | Flavonoid | -2.17 | 3 | Spectral match |
| 8.39 | 609.1460 | Quercetin -*O*-hexosyl-deoxyhexose | C_27_H_30_O_16_ | [M-H]^-^ | -0.2 | Flavonoid | 1.51 | 3 | Spectral match |
| 8.51 | 289.0720 | Epicatechin or catechin | C_15_H_14_O_6_ | [M-H]^-^ | 0.8 | Flavonoid | -3.08 | 3 | Spectral match |
| 8.67 | 449.1091 | Eriodictyol-*O*-hexose isomer | C_21_H_22_O_11_ | [M-H]^-^ | 0.4 | Flavonoid | -3.63 | 3 | GNPS |
| 9.39 | 449.1089 | Eriodictyol-*O*-hexose isomer | C_21_H_22_O_11_ | [M-H]^-^ | -0.1 | Flavonoid | -4.78 | 3 | GNPS |
| 9.65 | 395.1610 | Coumaroyl-caffeoyl-putrescine isomer | C_22_H_24_N_2_O_5_ | [M-H]^-^ | -0.6 | Phenolamide | 1.19 | 3 | GNPS |
| 9.95 | 435.1299 | Phloretin-*O*-hexoside isomer | C_21_H_24_O_10_ | [M-H]^-^ | 0.5 | Flavonoid | -4.33 | 3 | Pollen library |
| 10.41 | 630.2455 | Tricaffeoyl-spermidine isomer | C_34_H_37_N_3_O_9_ | [M-H]^-^ | -0.3 | Phenolamide | 4.72 | 3 | Manual |
| 11.36 | 639.3186 | Tricoumaroyl-spermine isomer | C_37_H_44_N_4_O_6_ | [M-H]^-^ | -0.3 | Phenolamide | 1.37 | 3 | Manual |
| 11.36 | 409.1771 | Coumaroyl-feruloyl putrescine isomer | C_23_H_26_N_2_O_5_ | [M-H]^-^ | 0.5 | Phenolamide | 3.09 | 3 | Pollen library |
| 11.74 | 744.3130 | Tricoumaroyl-caffeoyl-spermidine isomer | C_43_H_43_N_3_O_9_ | [M-H]^-^ | 27.3 | Phenolamide | -4.30 | 3 | GNPS |
| 12.21 | 614.2507 | Dicaffeoyl-coumaroyl-spermidine isomer | C_34_H_37_N_3_O_8_ | [M-H]^-^ | -0.1 | Phenolamide | 1.73 | 3 | Manual |
| 12.60 | 614.2509 | Dicaffeoyl-coumaroyl-spermidine isomer | C_34_H_37_N_3_O_8_ | [M-H]^-^ | 0.2 | Phenolamide | 1.88 | 3 | Manual |
| 12.88 | 598.2560 | Dicoumaroyl-caffeoyl-spermidine isomer | C_34_H_37_N_3_O_7_ | [M-H]^-^ | 0.2 | Phenolamide | 6.81 | 3 | Pollen library |
| 13.14 | 614.2514 | Dicaffeoyl-coumaroyl-spermidine isomer | C_34_H_37_N_3_O_8_ | [M-H]^-^ | 1.0 | Phenolamide | 1.16 | 3 | Manual |
| 13.14 | 630.2438 | Tricaffeoyl-spermidine isomer | C_34_H_37_N_3_O_9_ | [M-H]^-^ | -3.0 | Phenolamide | 1.27 | 3 | Pollen library |
| 13.15 | 1229.5078 | Coumaroyl-dicaffeoyl-spermidine isomer | C_34_H_37_N_3_O_8_ | [2M-H]^-^ | -0.9 | Phenolamide | 2.18 | 3 | Pollen library |
| 13.55 | 359.0774 | Rosmarinic acid | C_18_H_16_O_8_ | [M-H]^-^ | 0.4 | Organic acid | -3.43 | 3 | Pollen library |
| 14.44 | 628.2663 | Dicoumaroyl-hydroxyferuloyl-spermidine isomer | C_35_H_39_N_3_O_8_ | [M-H]^-^ | -0.2 | Phenolamide | 2.10 | 3 | Pollen library |
| 15.13 | 582.2609 | Tricoumaroyl-spermidine isomer | C_34_H_37_N_3_O_6_ | [M-H]^-^ | -0.1 | Phenolamide | 2.44 | 3 | Manual |
| 15.34 | 628.2664 | Dicoumaroyl-hydroxyferuloyl-spermidine isomer | C_35_H_39_N_3_O_8_ | [M-H]^-^ | -0.1 | Phenolamide | 1.72 | 3 | Pollen library |
| 15.36 | 1197.5180 | Dicoumaroyl-caffeoyl-spermidine isomer | C_34_H_37_N_3_O_7_ | [2M-H]^-^ | -0.9 | Phenolamide | 1.83 | 3 | Pollen library |
| 15.78 | 285.0401 | Luteolin | C_15_H_10_O_6_ | [M-H]^-^ | -1.3 | Flavonoid | -1.48 | 3 | Pollen library |
| 15.96 | 598.2560 | Dicoumaroyl-caffeoyl-spermidine isomer | C_34_H_37_N_3_O_7_ | [M-H]^-^ | 0.2 | Phenolamide | 1.40 | 3 | Pollen library |
| 16.25 | 1257.5391 | Dicoumaroyl-hydroxyferuloyl-spermidine isomer | C_35_H_39_N_3_O_8_ | [2M-H]^-^ | -0.8 | Phenolamide | 3.89 | 3 | Pollen library |
| 16.46 | 598.2558 | Dicoumaroyl-caffeoyl-spermidine isomer | C_34_H_37_N_3_O_7_ | [M-H]^-^ | -0.1 | Phenolamide | 1.77 | 3 | Pollen library |
| 16.69 | 565.1120 | Isoginkgetin | C_32_H_22_O_10_ | [M-H]^-^ | -3.6 | Flavonoid | -4.30 | 3 | GNPS |
| 16.70 | 285.0395 | Fisetin | C_15_H_10_O_6_ | [M-H]^-^ | -3.4 | Flavonoid | -3.31 | 3 | Pollen library |
| 16.76 | 658.2767 | Caffeoyl-diferuloyl-spermidine isomer | C_36_H_41_N_3_O_9_ | [M-H]^-^ | -0.5 | Phenolamide | 1.95 | 3 | Pollen library |
| 16.94 | 545.1420 | Phloretin derivative | C_30_H_26_O_10_ | [M-H]^-^ | -6.1 | Flavonoid | -3.20 | 3 | GNPS |
| 16.95 | 582.2610 | Tricoumaroyl-spermidine isomer | C_34_H_37_N_3_O_6_ | [M-H]^-^ | 0.1 | Phenolamide | 2.05 | 3 | Manual |
| 16.99 | 628.2664 | Dicoumaroyl-hydroxyferuloyl-spermidine isomer | C_35_H_39_N_3_O_8_ | [M-H]^-^ | -0.1 | Phenolamide | 1.42 | 3 | Pollen library |
| 17.15 | 301.0720 | Homoeriodictyol | C_16_H_14_O_6_ | [M-H]^-^ | 0.8 | Flavonoid | -1.18 | 2 | Spectral match |
| 17.53 | 612.2715 | Dicoumaroyl-feruloyl-spermidine isomer | C_35_H_39_N_3_O_7_ | [M-H]^-^ | 0.0 | Phenolamide | 2.62 | 3 | Pollen library |
| 17.76 | 582.2610 | Tricoumaroyl-spermidine isomer | C_34_H_37_N_3_O_6_ | [M-H]^-^ | 0.1 | Phenolamide | 1.63 | 3 | Manual |
| 18.29 | 628.2664 | Dicoumaroyl-hydroxyferuloyl-spermidine isomer | C_35_H_39_N_3_O_8_ | [M-H]^-^ | -0.1 | Phenolamide | 1.01 | 3 | Pollen library |
| 18.52 | 301.0720 | Hesperetin | C_16_H_14_O_6_ | [M-H]^-^ | 0.8 | Flavonoid | -4.40 | 2 | Spectral match |
| 19.17 | 658.2768 | Caffeoyl-diferuloyl-spermidine isomer | C_36_H_41_N_3_O_9_ | [M-H]^-^ | -0.3 | Phenolamide | -2.56 | 3 | Pollen library |
| 20.96 | 628.2661 | Dicoumaroyl-hydroxyferuloyl-spermidine isomer | C_35_H_39_N_3_O_8_ | [M-H]^-^ | -0.5 | Phenolamide | 2.24 | 3 | Pollen library |
| 21.55 | 1257.5387 | Dicoumaroyl-hydroxyferuloyl-spermidine isomer | C_35_H_39_N_3_O_8_ | [2M-H]^-^ | -1.2 | Phenolamide | 3.91 | 3 | Pollen library |
| 22.57 | 788.3390 | Phenolamide derivative | C_45_H_49_N_4_O_9_ | [M-H]^-^ | -4.7 | Phenolamide | -6.18 | 3 | GNPS |
| 22.98 | 612.2714 | Dicoumaroyl-feruloyl-spermidine isomer | C_35_H_39_N_3_O_7_ | [M-H]^-^ | -0.2 | Phenolamide | 3.01 | 3 | Manual |
| 23.09 | 672.2925 | Triferuloyl-spermidine isomer | C_37_H_43_N_3_O_9_ | [M-H]^-^ | -0.2 | Phenolamide | -2.79 | 3 | Pollen library |
| 25.89 | 612.2714 | Dicoumaroyl-feruloyl-spermidine isomer | C_35_H_39_N_3_O_7_ | [M-H]^-^ | -0.2 | Phenolamide | 2.34 | 3 | Manual |
| 28.84 | 555.1299 | Naringenin derivative | C_31_H_24_O_10_ | [M-H]^-^ | 0.4 | Flavonoid | -2.99 | 3 | Manual |
| 32.42 | 465.2261 | LPG 15:2 isomer | C_21_H_39_O_9_P | [M-H]^-^ | 0.4 | Lipid | 1.92 | 3 | Lipid module MZmine |
| 33.09 | 447.2159 | LPG O-15:4 isomer | C_21_H_37_O_8_P | [M-H]^-^ | 1.3 | Lipid | 2.18 | 3 | Lipid module MZmine |
| 33.51 | 447.2156 | LPG O-15:4 isomer | C_21_H_37_O_8_P | [M-H]^-^ | 0.6 | Lipid | 1.77 | 3 | Lipid module MZmine |
| 33.72 | 555.1299 | Naringenin cinnamic acid derivative | C_31_H_24_O_10_ | [M-H]^-^ | 0.4 | Flavonoid | -2.72 | 3 | Manual |
| 33.86 | 447.2156 | LPG O-15:4 isomer | C_21_H_37_O_8_P | [M-H]^-^ | 0.6 | Lipid | 2.33 | 3 | Lipid module MZmine |
| 34.47 | 409.0930 | Naringenin derivative | C_22_H_18_O_8_ | [M-H]^-^ | 0.3 | Flavonoid | -3.91 | 3 | Manual |
| 34.71 | 658.2806 | Caffeoyl-diferuloyl-spermidine isomer | C_36_H_41_N_3_O_9_ | [M-H]^-^ | 5.5 | Phenolamide | -3.74 | 3 | Pollen library |
| 35.03 | 449.2312 | LPG O-15:3 | C_21_H_39_O_8_P | [M-H]^-^ | 0.5 | Lipid | 1.30 | 3 | Lipid module MZmine |
| 35.41 | 447.2155 | LPG O-15:4 isomer | C_21_H_37_O_8_P | [M-H]^-^ | 0.4 | Lipid | 1.90 | 3 | Lipid module MZmine |
| 35.53 | 555.1301 | Naringenin cinnamic acid derivative | C_31_H_24_O_10_ | [M-H]^-^ | 0.8 | Flavonoid | -3.76 | 3 | Manual |
| 35.67 | 869.4320 | Naringenin derivative | C_47_H_66_O_15_ | [M-H]^-^ | -1.0 | Flavonoid | -4.95 | 3 | Manual |
| 35.72 | 465.2262 | LPG 15:2 isomer | C_21_H_39_O_9_P | [M-H]^-^ | 0.7 | Lipid | 4.84 | 3 | Lipid module MZmine |
| 35.80 | 449.2312 | LPG O-15:3 | C_21_H_39_O_8_P | [M-H]^-^ | 0.5 | Lipid | 1.71 | 3 | Lipid module MZmine |
| 35.84 | 569.1451 | Naringenin derivative | C_32_H_26_O_10_ | [M-H]^-^ | -0.4 | Flavonoid | -4.23 | 3 | Manual |
| 35.97 | 447.2156 | LPG O-15:4 isomer | C_21_H_37_O_8_P | [M-H]^-^ | 0.6 | Lipid | 2.12 | 3 | Lipid module MZmine |
| 36.19 | 781.3803 | Naringenin derivative | C_43_H_58_O_13_ | [M-H]^-^ | -0.2 | Flavonoid | -1.19 | 3 | Manual |
| 36.24 | 867.4169 | Naringenin derivative | C_47_H_64_O_15_ | [M-H]^-^ | -0.4 | Flavonoid | -1.48 | 3 | Manual |
| 36.35 | 313.2386 | Dihydroxy octadecenoic acid (FA 18:1;2O) | C_18_H_34_O_4_ | [M-H]^-^ | 0.5 | Lipid | 1.17 | 3 | Spectral match |
| 36.41 | 447.2156 | LPG O-15:4 isomer | C_21_H_37_O_8_P | [M-H]^-^ | 0.6 | Lipid | 1.65 | 3 | Lipid module MZmine |
| 37.02 | 850.4100 | Naringenin derivative | C_29_H_71_O_27_ | [M-H]^-^ | -1.2 | Flavonoid | -1.20 | 3 | GNPS |
| 37.03 | 569.1452 | Naringenin derivative | C_32_H_26_O_10_ | [M-H]^-^ | -0.2 | Flavonoid | -4.52 | 3 | Manual |
| 37.03 | 849.4067 | Naringenin derivative | C_47_H_62_O_14_ | [M-H]^-^ | 0.0 | Flavonoid | -1.15 | 3 | Manual |
| 37.06 | 555.1299 | Naringenin cinnamic acid derivative | C_31_H_24_O_10_ | [M-H]^-^ | 0.4 | Flavonoid | -3.82 | 3 | Manual |
| 37.23 | 805.3802 | Naringenin derivative | C_45_H_58_O_13_ | [M-H]^-^ | -0.3 | Flavonoid | -1.36 | 3 | Manual |
| 37.25 | 761.3539 | Naringenin derivative | C_43_H_54_O_12_ | [M-H]^-^ | -0.5 | Flavonoid | 1.18 | 3 | Manual |
| 37.38 | 474.2625 | LPE 18:3 | C_23_H_42_NO_7_P | [M-H]^-^ | -0.2 | Lipid | 1.60 | 3 | Lipid module MZmine |
| 37.46 | 299.0563 | Hydroxygenistein-*O*-methyl isomer | C_16_H_12_O_6_ | [M-H]^-^ | 0.6 | Flavonoid | -4.23 | 3 | Pollen library |
| 37.83 | 849.4064 | Naringenin derivative | C_47_H_62_O_14_ | [M-H]^-^ | -0.3 | Flavonoid | -1.55 | 3 | Manual |
| 38.10 | 505.2573 | LPG 18:3 | C_24_H_43_O_9_P | [M-H]^-^ | 0.2 | Lipid | 1.69 | 3 | Lipid module MZmine |
| 38.38 | 805.3801 | Naringenin derivative | C_45_H_58_O_13_ | [M-H]^-^ | -0.5 | Flavonoid | -1.67 | 3 | Manual |
| 38.53 | 299.0563 | Hydroxygenistein-*O*-methyl isomer | C_16_H_12_O_6_ | [M-H]^-^ | 0.6 | Flavonoid | -2.87 | 3 | Pollen library |
| 38.56 | 431.2207 | LPA 18:3 | C_21_H_37_O_7_P | [M-H]^-^ | 0.7 | Lipid | 1.46 | 3 | Lipid module MZmine |
| 38.56 | 499.2077 | LPG 18:6 | C_24_H_37_O_9_P | [M-H]^-^ | -5.1 | Lipid | 3.14 | 3 | Lipid module MZmine |
| 38.69 | 381.2050 | LPA 14:0 | C_17_H_35_O_7_P | [M-H]^-^ | 0.6 | Lipid | 2.28 | 3 | Lipid module MZmine |
| 38.72 | 476.2784 | LPE 18:2 | C_23_H_44_NO_7_P | [M-H]^-^ | 0.3 | Lipid | 1.81 | 2 | Spectral match |
| 39.00 | 499.2080 | LPG 18:6 | C_24_H_37_O_9_P | [M-H]^-^ | -4.5 | Lipid | 2.41 | 3 | Lipid module MZmine |
| 39.77 | 507.2731 | LPG 18:2 | C_24_H_45_O_9_P | [M-H]^-^ | 0.5 | Lipid | 1.85 | 2 | Spectral match |
| 40.27 | 433.2363 | LPA 18:2 | C_21_H_39_O_7_P | [M-H]^-^ | 0.5 | Lipid | 1.55 | 3 | Lipid module MZmine |
| 40.27 | 501.2237 | LPG 18:5 | C_24_H_39_O_9_P | [M-H]^-^ | -4.4 | Lipid | 2.63 | 3 | Lipid module MZmine |
| 40.37 | 483.2730 | LPG 16:0 isomer | C_22_H_45_O_9_P | [M-H]^-^ | 0.3 | Lipid | 1.08 | 3 | Lipid module MZmine |
| 40.39 | 395.2207 | LPA 15:0 | C_18_H_37_O_7_P | [M-H]^-^ | 0.7 | Lipid | 1.66 | 3 | Lipid module MZmine |
| 40.75 | 501.2236 | LPG 18:5 | C_24_H_39_O_9_P | [M-H]^-^ | -4.6 | Lipid | 2.52 | 3 | Lipid module MZmine |
| 41.06 | 483.2733 | LPG 16:0 isomer | C_22_H_45_O_9_P | [M-H]^-^ | 0.9 | Lipid | 1.17 | 3 | Manual |
| 41.47 | 483.2730 | LPG 16:0 isomer | C_22_H_45_O_9_P | [M-H]^-^ | 0.3 | Lipid | 2.25 | 3 | Manual |
| 41.56 | 409.2363 | LPA 16:0 | C_19_H_39_O_7_P | [M-H]^-^ | 0.6 | Lipid | 1.41 | 3 | Lipid module MZmine |
| 43.06 | 435.2519 | LPA 18:1 | C_21_H_41_O_7_P | [M-H]^-^ | 0.4 | Lipid | 1.78 | 3 | Lipid module MZmine |
| **C18 (+)** | | | | | | | | | |
| 3.22 | 251.1391 | Caffeoyl putrescin | C_13_H_18_N_2_O_3_ | [M+H]^+^ | 0.3 | Phenolamide | -1.17 | 2 | Spectral match |
| 3.72 | 330.2025 | Hetisine-like alkaloid | C_20_H_27_NO_3_ | [M+H]^+^ | -11.7 | Alkaloid | 1.51 | 3 | Spectral match |
| 5.06 | 277.1659 | Coumaroyl agmatine | C_14_H_20_N_4_O_2_ | [M+H]^+^ | 0.0 | Phenolamide | 2.38 | 2 | Spectral match |
| 5.26 | 773.2140 | Quercetin-*O*-hexosyl-hexose-*O*-deoxyhexose | C_33_H_40_O_21_ | [M+H]^+^ | 0.7 | Flavonoid | 3.44 | 3 | GNPS |
| 5.45 | 279.1700 | Feruloyl cadaverine | C_15_H_22_N_2_O_3_ | [M+H]^+^ | -1.1 | Phenolamide | 3.68 | 3 | GNPS |
| 5.60 | 757.2183 | Kaempferol-*O*-hexosyl-deoxyhexose-*O*-hexose | C_33_H_40_O_20_ | [M+H]^+^ | -0.4 | Flavonoid | 4.65 | 3 | Pollen library |
| 6.27 | 454.2340 | Caffeoyl-coumaroyl-spermidine isomer | C_25_H_31_N_3_O_5_ | [M+H]^+^ | 0.8 | Phenolamide | 1.54 | 3 | GNPS |
| 6.53 | 470.2280 | Dicaffeoyl-spermidine | C_25_H_31_N_3_O_6_ | [M+H]^+^ | -1.2 | Phenolamide | -1.29 | 3 | GNPS |
| 6.69 | 597.1450 | Quercetin-*O*-hexosyl-pentose | C_26_H_28_O_16_ | [M+H]^+^ | 0.0 | Flavonoid | 2.07 | 3 | GNPS |
| 7.23 | 264.1960 | Dendrobine-like alkaloid | C_16_H_25_NO_2_ | [M+H]^+^ | 0.7 | Alkaloid | 6.98 | 3 | Spectral match |
| 7.31 | 595.1658 | Kaempferol-*O*-hexosyl-deoxyhexose | C_27_H_30_O_15_ | [M+H]^+^ | 0.1 | Flavonoid | -1.58 | 3 | Spectral match |
| 7.35 | 773.1920 | Quercetin-*O*-coumaroyl-hexose-*O*-hexose | C_36_H_36_O_19_ | [M+H]^+^ | -0.5 | Flavonoid | -4.14 | 3 | GNPS |
| 7.51 | 451.1233 | Eriodictyol-*O*-hexose | C_21_H_22_O_11_ | [M+H]^+^ | -0.4 | Flavonoid | -3.23 | 3 | Spectral match |
| 7.57 | 287.0560 | Fisetin | C_15_H_10_O_6_ | [M+H]^+^ | 3.4 | Flavonoid | -1.87 | 2 | Spectral match |
| 7.58 | 453.2571 | Hydroxy-jasmonic-acid | C_12_H_18_O_4_ | [2M+H]^+^ | 19.4 | Jasmonate / Plant Hormone | 2.43 | 3 | Pollen library |
| 8.34 | 285.0758 | Methylgenistein | C_16_H_12_O_5_ | [M+H]^+^ | 0.2 | Flavonoid | -1.43 | 2 | Spectral match |
| 8.45 | 498.2603 | Diferuloyl-spermidine isomer | C_27_H_35_N_3_O_6_ | [M+H]^+^ | 0.9 | Phenolamide | -1.62 | 3 | Pollen library |
| 8.91 | 435.1293 | Naringenin-*O*-hexose | C_21_H_22_O_10_ | [M+H]^+^ | 1.7 | Flavonoid | -4.43 | 3 | Spectral match |
| 9.08 | 498.2600 | Diferuloyl-spermidine isomer | C_27_H_35_N_3_O_6_ | [M+H]^+^ | 0.3 | Phenolamide | -2.13 | 3 | Pollen library |
| 9.65 | 468.2490 | Coumaroyl-feruloyl-spermidine isomer | C_26_H_33_N_3_O_5_ | [M+H]^+^ | -0.6 | Phenolamide | -1.60 | 3 | GNPS |
| 10.12 | 413.1787 | Dicaffeoyl-putrescine isomer | C_22_H_24_N_2_O_6_ | [M+H]^+^ | 19.3 | Phenolamide | 2.68 | 3 | Pollen library |
| 10.62 | 435.1290 | Naringenin-*O*-hexose | C_21_H_22_O_10_ | [M+H]^+^ | 1.0 | Flavonoid | -1.47 | 3 | GNPS |
| 10.89 | 632.2600 | Tricaffeoyl-spermidine isomer | C_34_H_37_N_3_O_9_ | [M+H]^+^ | -0.4 | Phenolamide | 2.10 | 3 | GNPS |
| 11.23 | 271.0600 | Genistein | C_15_H_10_O_5_ | [M+H]^+^ | -0.4 | Flavonoid | -1.91 | 2 | GNPS |
| 11.41 | 470.2290 | Dicaffeoyl-spermidine isomer | C_25_H_31_N_3_O_6_ | [M+H]^+^ | 0.9 | Phenolamide | 1.19 | 3 | GNPS |
| 11.41 | 632.2600 | Tricaffeoyl-spermidine isomer | C_34_H_37_N_3_O_9_ | [M+H]^+^ | -0.4 | Phenolamide | 1.20 | 3 | GNPS |
| 12.52 | 289.0707 | Eriodictyol | C_15_H_12_O_6_ | [M+H]^+^ | 0.1 | Flavonoid | -2.32 | 2 | Spectral match |
| 12.70 | 303.0499 | Quercetin | C_15_H_10_O_7_ | [M+H]^+^ | -0.1 | Flavonoid | -1.26 | 2 | Spectral match |
| 12.70 | 287.0550 | Kaempferol | C_15_H_10_O_6_ | [M+H]^+^ | -0.1 | Flavonoid | -1.03 | 2 | Spectral match |
| 12.82 | 616.2655 | Dicaffeoyl-coumaroyl-spermidine isomer | C_34_H_37_N_3_O_8_ | [M+H]^+^ | 0.3 | Phenolamide | 1.16 | 3 | Pollen library |
| 12.93 | 776.3390 | Dicoumaroyl-caffeoyl-feruloyl-spermidine isomer | C_44_H_45_N_3_O_10_ | [M+H]^+^ | 27.3 | Phenolamide | -2.71 | 3 | GNPS |
| 13.10 | 1231.5220 | Dicaffeoyl-coumaroyl-spermidine isomer | C_34_H_37_N_3_O_8_ | [2M+H]^+^ | -1.1 | Phenolamide | 2.65 | 3 | Pollen library |
| 13.41 | 600.2700 | Dicoumaroyl-caffeoyl-spermidine isomer | C_34_H_37_N_3_O_7_ | [M+H]^+^ | -0.7 | Phenolamide | 2.51 | 3 | GNPS |
| 14.03 | 600.2700 | Dicoumaroyl-caffeoyl-spermidine isomer | C_34_H_37_N_3_O_7_ | [M+H]^+^ | -0.7 | Phenolamide | 1.41 | 3 | GNPS |
| 14.68 | 447.1891 | Crosatoside B | C_20_H_30_O_11_ | [M+H]^+^ | 6.7 | Terpenoid | 4.38 | 3 | Pollen library |
| 14.89 | 1199.5322 | Dicoumaroyl-caffeoyl-spermidine isomer | C_34_H_37_N_3_O_7_ | [2M+H]^+^ | -1.2 | Phenolamide | 2.47 | 3 | Pollen library |
| 14.90 | 632.2596 | Tricaffeoyl-spermidine isomer | C_34_H_37_N_3_O_9_ | [M+H]^+^ | -1.0 | Phenolamide | 1.22 | 3 | Pollen library |
| 15.26 | 1199.5323 | Dicoumaroylcaffeoyl-spermidine isomer | C_34_H_37_N_3_O_7_ | [2M+H]^+^ | -1.1 | Phenolamide | 2.93 | 3 | Pollen library |
| 15.27 | 632.2599 | Tricaffeoyl-spermidine isomer | C_34_H_37_N_3_O_9_ | [M+H]^+^ | -0.6 | Phenolamide | 1.12 | 3 | Pollen library |
| 15.28 | 630.2807 | Dicoumaroyl-hydroxyferuloyl spermidine isomer | C_35_H_39_N_3_O_8_ | [M+H]^+^ | -0.5 | Phenolamide | 2.38 | 3 | Pollen library |
| 16.01 | 584.2750 | Tricoumaroyl-spermidine isomer | C_34_H_37_N_3_O_6_ | [M+H]^+^ | -0.9 | Phenolamide | 2.34 | 3 | Spectral match |
| 16.17 | 630.2808 | Dicoumaroyl-hydroxyferuloyl spermidine isomer | C_35_H_39_N_3_O_8_ | [M+H]^+^ | -0.3 | Phenolamide | 1.79 | 3 | Pollen library |
| 16.20 | 468.2490 | Coumaroyl-feruloyl-spermidine isomer | C_26_H_33_N_3_O_5_ | [M+H]^+^ | -0.6 | Phenolamide | 1.71 | 3 | GNPS |
| 16.54 | 273.0764 | Naringenin | C_15_H_12_O_5_ | [M+H]^+^ | 2.4 | Flavonoid | -2.84 | 2 | Spectral match |
| 16.88 | 275.0904 | Phloretin (Dihydronaringenin) | C_15_H_14_O_5_ | [M+H]^+^ | -3.6 | Flavonoid | -1.46 | 3 | Pollen library |
| 17.52 | 584.2750 | Tricoumaroyl-spermidine isomer | C_34_H_37_N_3_O_6_ | [M+H]^+^ | -0.9 | Phenolamide | 2.07 | 3 | GNPS |
| 17.92 | 273.0757 | Naringenin chalcone | C_15_H_12_O_5_ | [M+H]^+^ | -0.2 | Flavonoid | -2.78 | 2 | Spectral match |
| 18.18 | 895.3843 | Dicaffeoyl-feruloyl-hydroxyferuloyl-spermine isomer | C_48_H_54_N_4_O_13_ | [M+H]^+^ | 0.09 | Phenolamide | 1.84 | 3 | Pollen library |
| 18.18 | 1167.5427 | Tricoumaroyl-spermidine isomer | C_34_H_37_N_3_O_6_ | [2M+H]^+^ | -0.9 | Phenolamide | 2.70 | 3 | Pollen library |
| 19.05 | 660.2913 | Diferuloyl-caffeoyl-spermidine isomer | C_36_H_41_N_3_O_9_ | [M+H]^+^ | -0.4 | Phenolamide | -3.12 | 3 | Pollen library |
| 23.43 | 614.2859 | Dicoumaroyl-feruloyl-spermidine isomer | C_35_H_39_N_3_O_7_ | [M+H]^+^ | -0.3 | Phenolamide | 2.25 | 3 | Pollen library |
| 26.51 | 454.2330 | Caffeoyl-coumaroyl-spermidine isomer | C_25_H_31_N_3_O_5_ | [M+H]^+^ | -1.4 | Phenolamide | 1.22 | 3 | GNPS |
| 26.53 | 646.2754 | Dicaffeoyl-feruloyl-spermidine isomer | C_35_H_39_N_3_O_9_ | [M+H]^+^ | -0.8 | Phenolamide | 2.08 | 3 | Pollen library |
| 26.54 | 1227.5636 | Dicoumaroyl-feruloyl-spermidine isomer | C_35_H_39_N_3_O_7_ | [2M+H]^+^ | -1.0 | Phenolamide | 4.64 | 3 | Pollen library |
| 28.04 | 614.2859 | Dicoumaroyl-feruloyl-spermidine isomer | C_35_H_39_N_3_O_7_ | [M+H]^+^ | -0.3 | Phenolamide | 1.45 | 3 | Pollen library |
| 30.35 | 598.2910 | Spermidine derivative | C_35_H_39_N_3_O_6_ | [M+H]^+^ | -0.3 | Phenolamide | 1.94 | 3 | GNPS |
| 36.54 | 318.3006 | Phytosphingosine | C_18_H_39_NO_3_ | [M+H]^+^ | 1.0 | Lipid | -2.35 | 3 | Spectral match |

**Supplementary figures**

**Figure S1. *Petunia hybrida* V26 and W115 plant growing and harvesting.** Plant experiments were conducted at the Leibniz Institute of Vegetable and Ornamental Crops (IGZ) located in Großbeeren, Brandenburg, Germany (52°22′N, 13°18′E, alt. 40 m).

**Figure S2. UHPLC-HRMS/MS base peak chromatograms of *Petunia hybrida* pollen non-polar extract using C8 column.** Pooled quality control (QC) samples of *P. hybrida* W115 and V26 pollen non-polar extract using C8 column in negative (-) and positive (+) ionisation modes.

**Figure S3. UHPLC-HRMS/MS base peak chromatograms of *Petunia hybrida* pollen non-polar extract using C18 column.** Pooled quality control (QC) samples of *P. hybrida* W115 and V26 pollen semi-polar extract using C18 column in negative (-) and positive (+) ionisation modes.

**Figure S4. UHPLC-HRMS/MS base peak chromatograms of *Petunia hybrida* pollen polar extract using HILIC column.** Pooled quality control (QC) samples of *P. hybrida* W115 and V26 pollen polar extract using HILIC column in negative (-) and positive (+) ionisation modes.

**Figure S5. Sunburst plot of CANOPUS predicted compound classes in *Petunia hybrida* pollen extracts.** Samples analysed using three different chromatographic columns (C8, C18, and HILIC) and two ionisation modes: (+) positive; (-) negative. Chemical classes structured hierarchically in accordance with the NPClassifier ontology. Individual chart sizes are proportional to the area of all classified features in each dataset.

**Figure S6. Volcano plots of *Petunia hybrida* pollen extrac**ts. Samples analysed using three different chromatographic columns (C8, C18, and HILIC) and two ionisation modes: (+) positive; (-) negative. The comparison direction is set as the ratio between V26/W115, where red dots represent features with higher abundance in pollen from violet flowers (V26), and the purple dots represent features with higher abundance in pollen from white flowers (W115).

**Figure S7.** Hierarchical clustering heatmap of Pearson correlation coefficients for flavonoid and phenolamides annotated using CANOPUS chemical class prediction in *P. hybrida* pollen extract analysed by UHPLC-HRMS using C18 column in negative and positive ionisation modes. Features are labelled 'F/' and 'P/', corresponding to flavonoids and phenolamides, respectively, followed by *m/z* and retention time.

**Figure S8. Individual sunburst plots of CANOPUS predicted compound classes in *Petunia hybrida* pollen extracts of two lines, V26 and W115, with differently coloured flowers.** Data analysed using UHPLC-HRMS using three different chromatographic columns (C8, C18, and HILIC) and two ionisation modes: (+) positive; (-) negative. Chemical classes structured hierarchically in accordance with the NPClassifier ontology. Individual slice sizes are proportional to the summed peak area of all classified features and arranged in descending order, clockwise.

**Figure S9. Distribution of metabolic shifts for the** **significantly discriminating metabolites listed in Table S11.** The x-axis indicates chemical classes and the y-axis indicates the log_2_ (Fold change). The fold change (FC) direction is set as the ratio between V26/W115, where positive values indicate higher abundance in pollen from violet flowers (V26), and negative values indicate higher abundance in pollen from white flowers (W115). Central boxes represent the middle 50% of the metabolites in that class (the interquartile range). Horizontal lines inside the boxes represent the median, which is the exact middle value of the dataset. The "x" marker inside the box represents the mean, which is the calculated average value of all metabolites in that class. The vertical lines extending from the boxes (whiskers) represent the minimum and maximum fold change values observed within each class, while individual points represent outliers or metabolites with extreme fold changes that fall outside the interquartile range. Phenolamide (blue) and Flavonoid (red) classes are highlighted to maintain visual consistency with the correlation heatmaps.

**Supplementary text**

**Text S1. MS2 spectral libraries used for compound annotation.**

- **MS-DIAL metabolomics MSP spectral kit**

<https://systemsomicslab.github.io/compms/msdial/main.html#MSP>

**Libraries** (07/2025): MassBank, ReSpect, Riken IMS oxidized phospholipds, GNPS, Fiehn HILIC, CASMI, MetaboBASE, RIKEN PlaSMA, Karolinska institute and Gunma zic-HILIC (DIA), Fiehn Pathogen Box, Fiehn/Vaniya natural product library, BMDMS-NP, PFAS, LipidBlast libraries (v. 68).

**Content**: Spectra from more than 16,000 and 8,000 unique compounds in ESI+ and in ESI-, respectively, from authentic standards.

- **MassBank of North America**

<http://mona.fiehnlab.ucdavis.edu/downloads>

**Content libraries** (07/2025): more than 235,000 experimental spectra, and more than 1.8 million i*n-silico* spectra, from 48 different libraries.

- **GNPS public libraries**

<https://gnps.ucsd.edu/ProteoSAFe/libraries.jsp>

**Content libraries** (07/2025): FDA libraries, Phytochemical library, NIH collections, Faulkner legacy, EMBL Metabolomics Core Facility, Pesticides, Medicines for Malaria Venture Pathogen Box, LDB Lichen DB, GNPS collections (NIST 14, miscellaneous, bile acids, Sigma’s metabolite library), Dereplicator, PNNL lipids, MIADB (monoterpene indole alkaloids), IQAMDB (isoquinolines), Sam Sik Kang legacy, HCE cell lysate lipids, IOBA-NHC, Nutri-Metabolomics, Sciex library, Berkeley lab, IIMN-based library, MassBank libraries, ReSpect, HMDB, CASMI, Sumner library. In total, 1,519,477 spectra records.

**Text S2. Prompts for AI-Assisted compound annotation curation.**

The following prompts were provided to the Google Gemini Pro model to perform the curation and classification of the putative compound annotations. The process was sequential.

**Prompt 1: Initial Analysis and Classification**

“I have a compound list annotation from an experiment of *Petunia hybrida* pollen extract using LC-HRMS. The annotations are from spectral similarity using publicly available spectral libraries using MZmine software, and also through FBMN at GNPS using analogue search by modified cosine algorithm. In addition, annotations are also from compound databases using monoisotopic mass. Help me in revising these annotations and classifying them. I'm looking mainly for misleading annotations of synthetic compounds and microbial compounds, and also artefacts.

For each unique compound, please perform the following:

Classification: Assign a chemical or biosynthetic class (e.g., Flavonoid, Alkaloid, Lipid, Amino Acid).

Verdict: Assign a verdict on its plausibility in a *Petunia hybrida* pollen sample, using only one of these three categories: 'Highly Plausible', 'Possible but Requires Scrutiny', or 'Nonsensical (False Positive)'.

Here is the list of compounds: *[Paste your full, raw compound list here]*”

**Prompt 2 (if applicable, for formatting): Final Output Generation**

“Format the complete analysis as a CSV file with " " (double quotes) separating the entries and the following columns: 'Compound', 'Classification', and 'Verdict'.”
